# Supplementary figures and images for: Genomic evidence reveals three W-autosome fusions in Heliconius butterflies
Source: PLoS Genet. 2024 Jul 18;20(7):e1011318. doi: 10.1371/journal.pgen.1011318 (PMC11257349; doi:10.1371/journal.pgen.1011318)

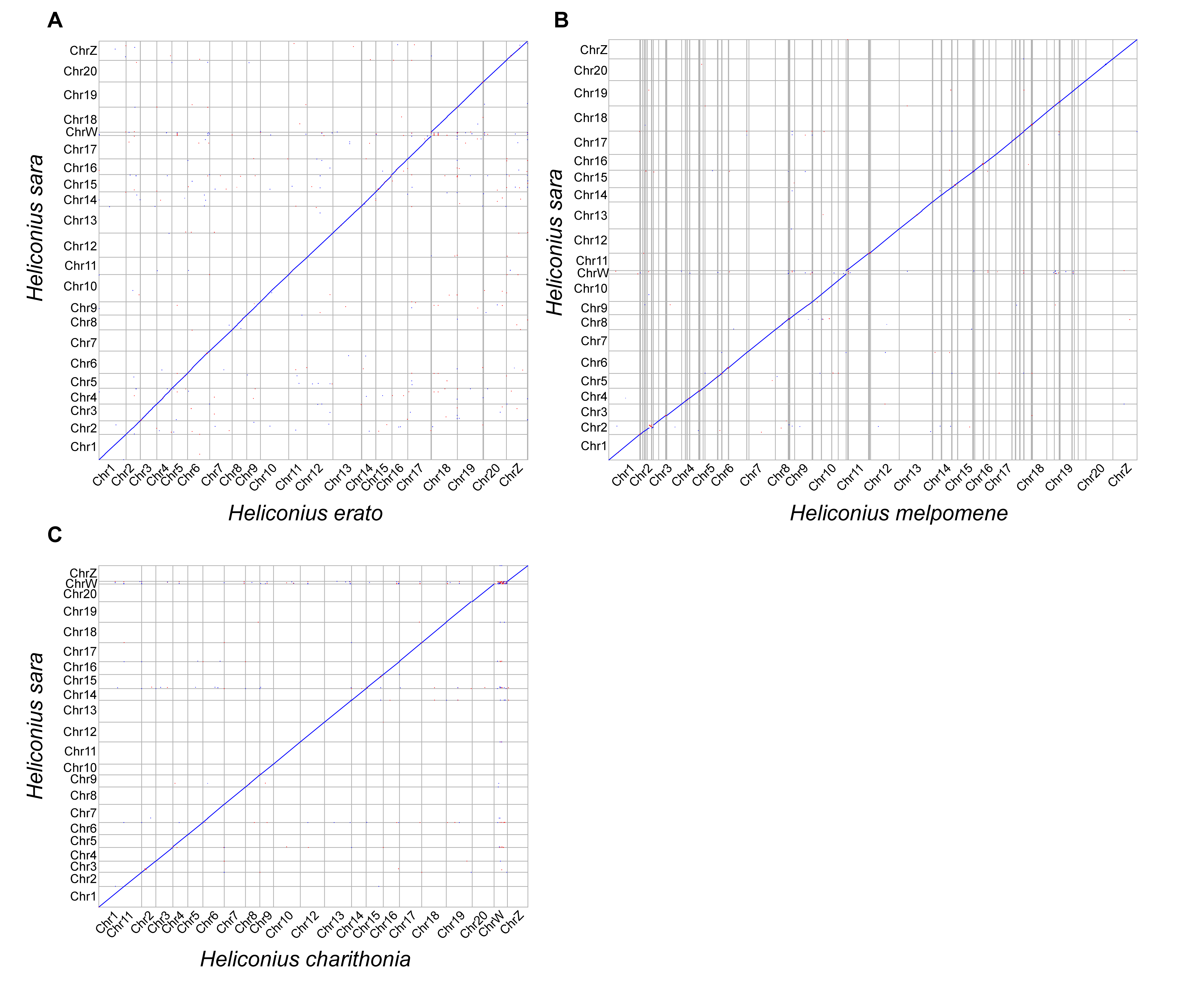

Supplement: S1 Fig — Pairwise alignment between chromosomes of (A) H. sara and H. erato, (B) H. sara and H. melpomene, and (C) H. sara and H. charithonia. The GenBank accession numbers for the genomes of H. melpomene, H. erato and H. charithonia are GCA_000313835.2, GCA_018249695.1 and GCA_030704555.1, respectively. The W chromosome in H. sara corresponds to a single homolog in H. charithonia. Because the W chromosome was not assembled in the genomes of H. erato and H. melpomene genomes, we could not compare the W of H. sara W against them. (TIF) [file pgen.1011318.s004.tif]

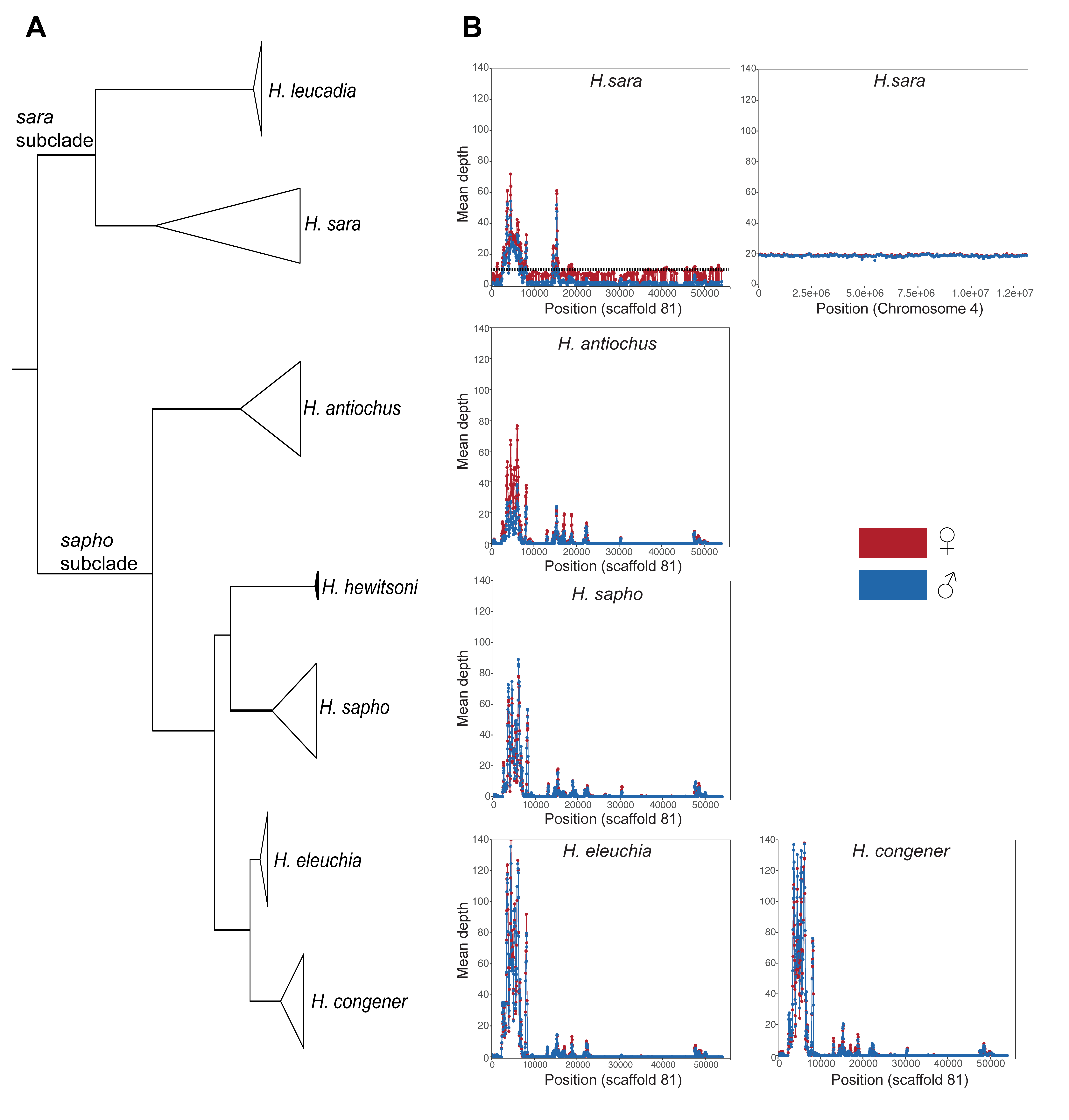

Supplement: S2 Fig — (A) Genome-wide topology of the sara-sapho clade. (B) Mean depth vs. position along the scaffold in each species. We plotted one (scaffold 81) out of the 32 scaffolds where females of the species H. sara showed half the sequencing depth of autosomes and where males do not map. The mean depth of an autosomal chromosome (Chr4) of the species H. sara is provided as an example for comparison with scaffold 81. Females are shown in red and males in blue. (TIF) [file pgen.1011318.s005.tif]

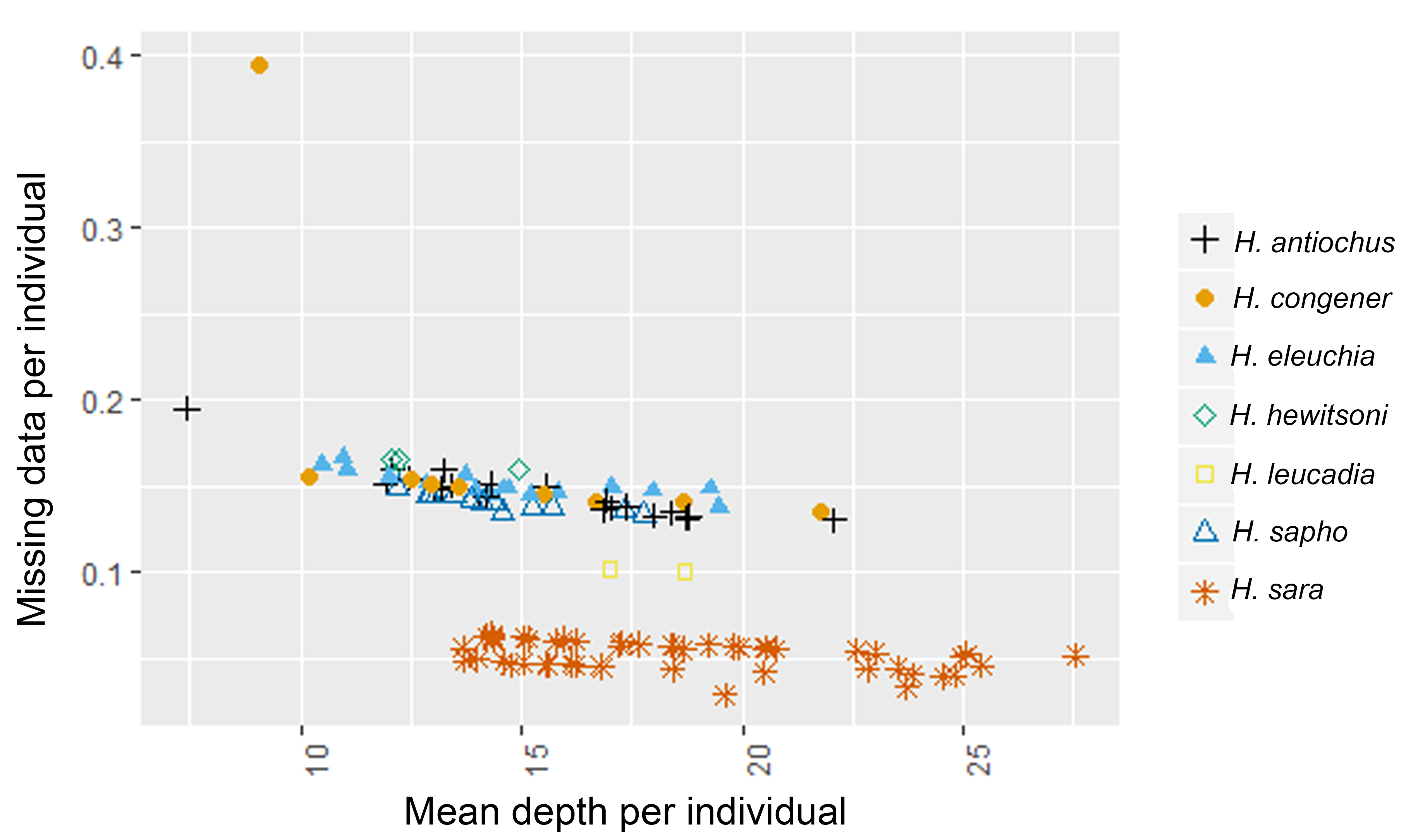

Supplement: S3 Fig — Each species is symbolised by a unique symbol and colour. Note that the lower missing data proportion in H. sara is likely due to its similarity with the reference genome (H. sara female). (TIF) [file pgen.1011318.s006.tif]

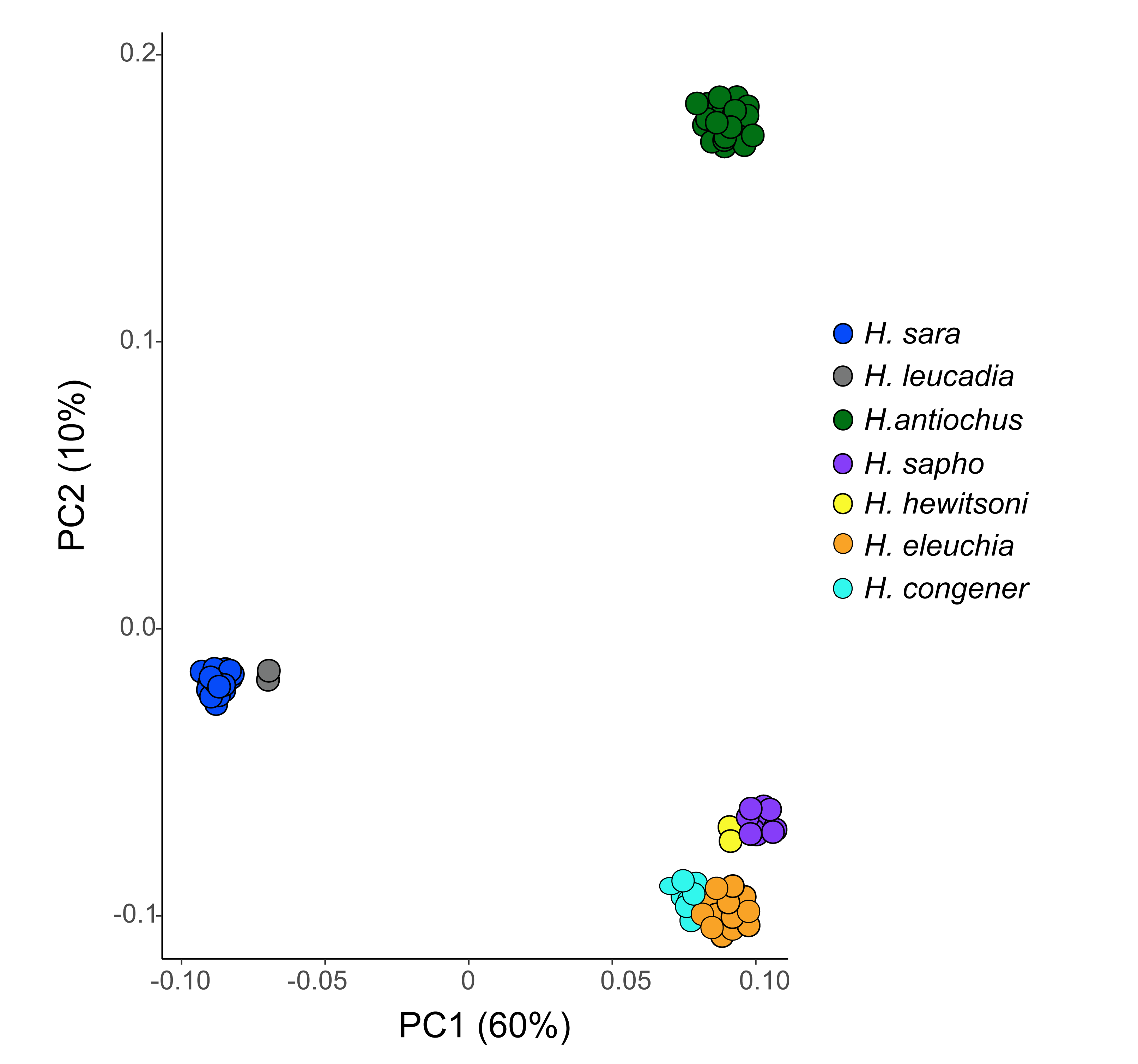

Supplement: S4 Fig — The PCA groups the individuals into two main groups: (i) H. sara and leucadia (hereafter sara subclade), and (ii) H. antiochus, H. eleuchia, H. congener, H. sapho and H. hewitsoni (hereafter sapho subclade). The first two principal components explain 60% (PC1) and 10% (PC2) of the total variance, respectively. PC1 separates the sara subclade from the sapho subclade, whereas PC2 separates H. antiochus from the rest of the species of the sapho subclade. H. sapho is closer to H. hewitsoni, whereas H. eleuchia is closer to H. congener. (TIF) [file pgen.1011318.s007.tif]

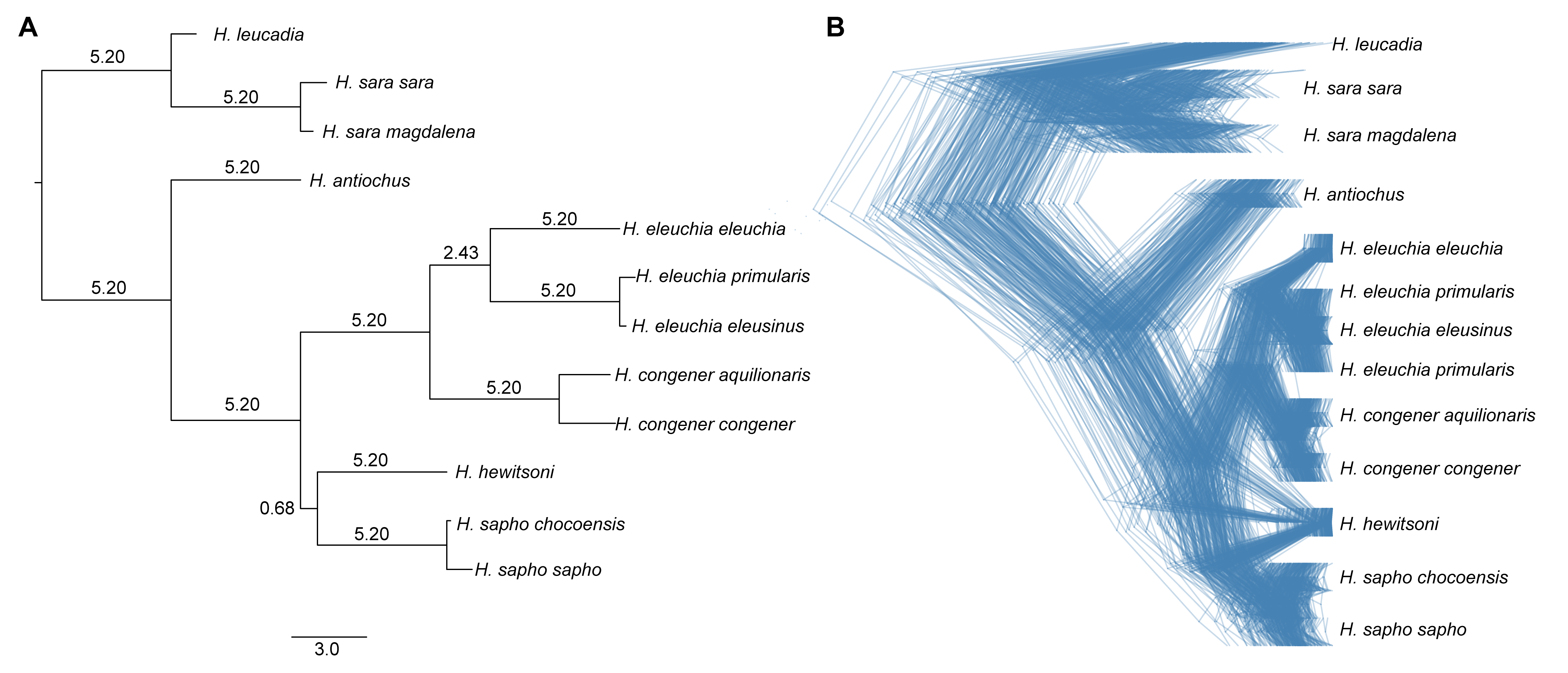

Supplement: S5 Fig — (A) ASTRAL species tree based on 271 phylogenetic trees (each recovered from a block of 100 loci). Branch lengths are shown in coalescent units. All nodes are supported with a bootstrap value of 100%. (B) DensiTree calculated from 271 topologies showing phylogenetic discordance within the sara-sapho clade. (TIF) [file pgen.1011318.s008.tif]

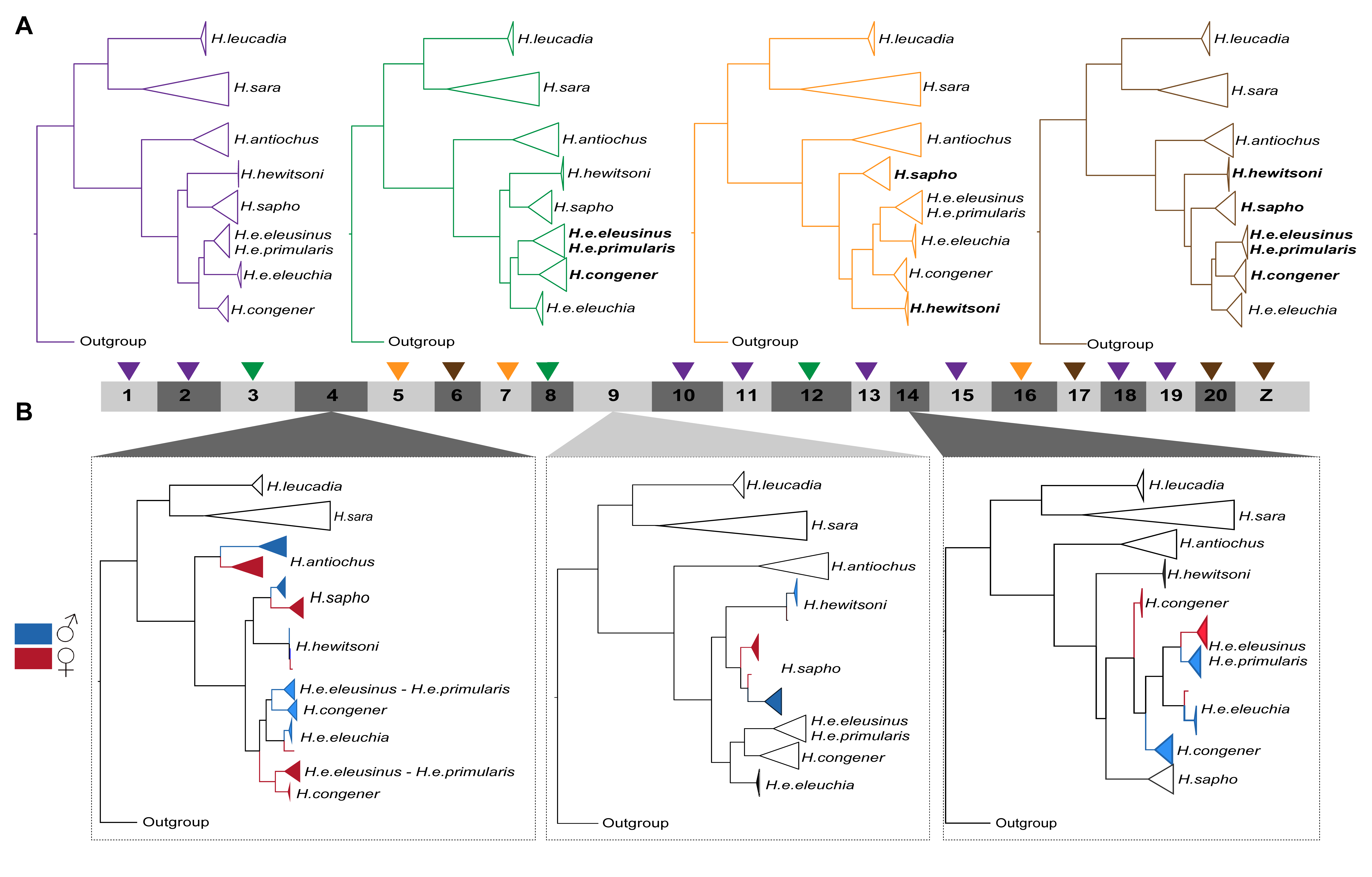

Supplement: S27 Fig — (A) Topologies found across the genome. Purple: genome-wide topology. Green: H. congener within H. eleuchia. Orange: H. hewitsoni as sister to H. congener + H. eleuchia. Brown: H. hewitsoni as sister to H. congener + H. eleuchia + H. sapho. Chromosomes are shown in the bottom, with coloured triangles indicating the topology revealed by each of them. (B) Topologies showing sex-specific grouping within some species, which is indicative of Sex-A fusions in Chr4, Chr9 and Chr14. In these species, females are coloured in red and males in blue. (TIF) [file pgen.1011318.s030.tif]

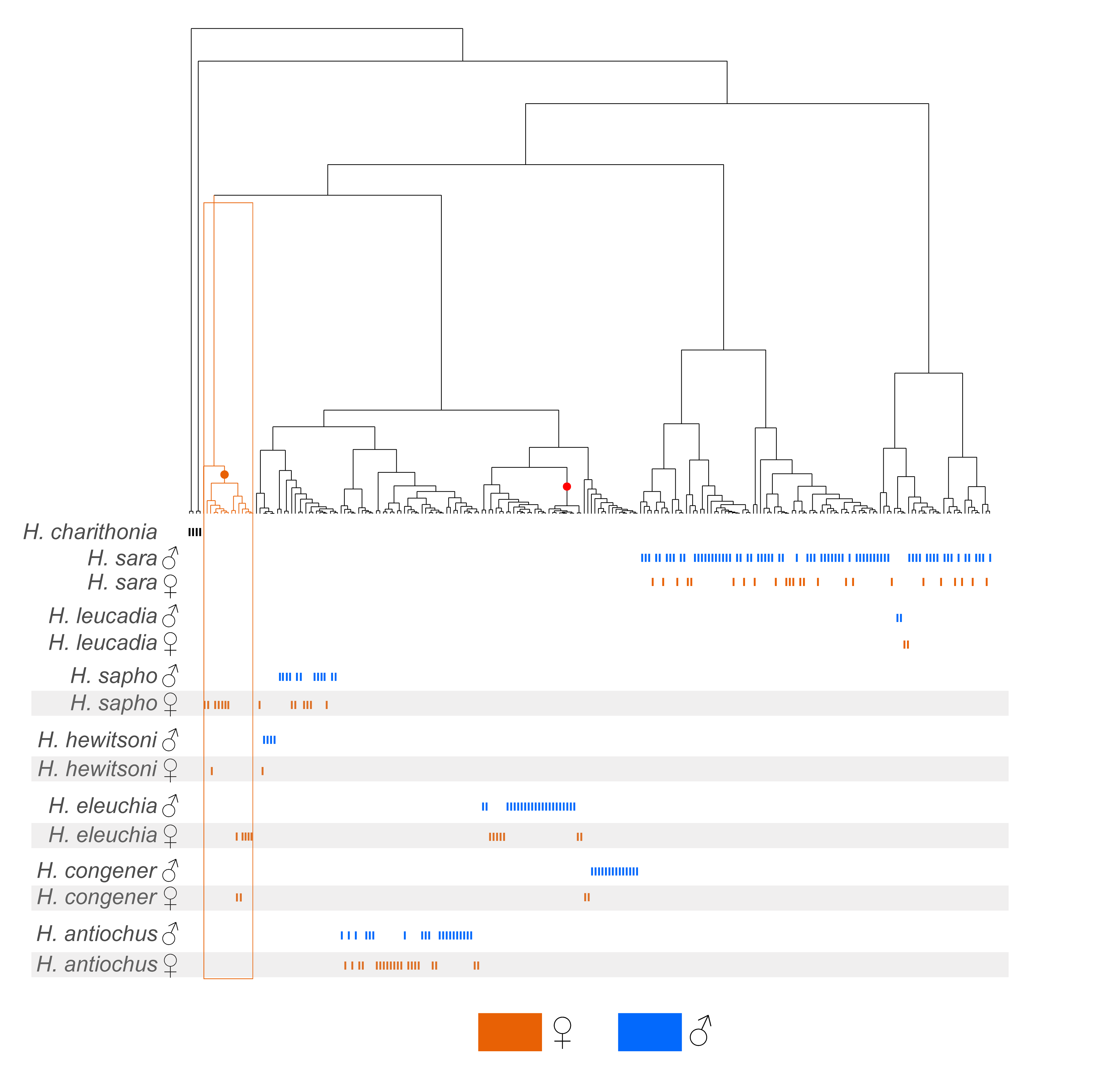

Supplement: S28 Fig — (A) This is one of the 112 marginal phylogenies where female alleles from at least two species in the sapho subclade cluster as expected in a Sex-A fusion. Each vertical line represents an individual allele, and the alleles of all individuals are shown differentiating those of females (orange) from those of males (blue). Note that one allele of the females clusters with the alleles of males, while the other female allele formed a separate group (highlighted in orange). (TIF) [file pgen.1011318.s031.tif]

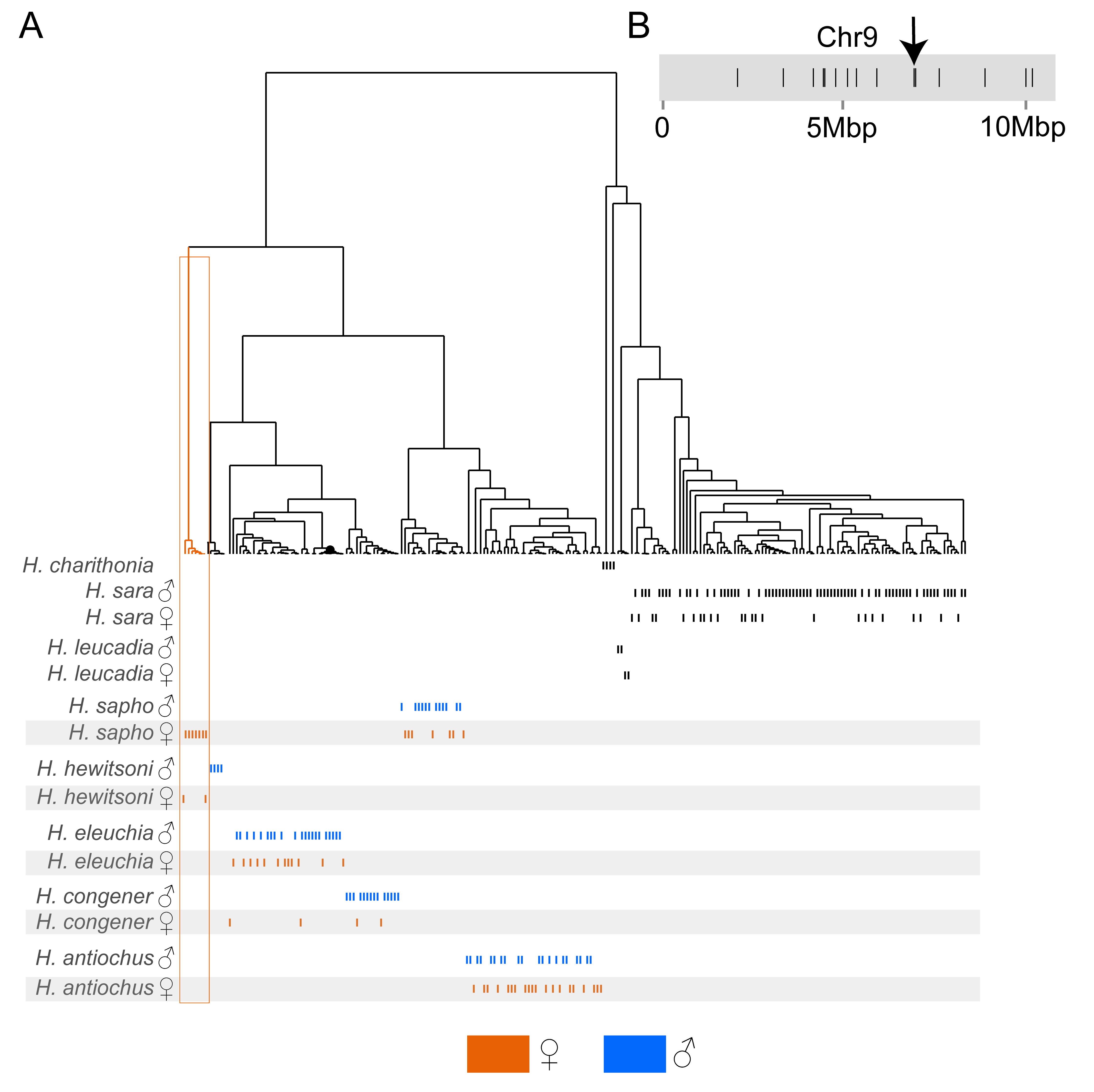

Supplement: S29 Fig — (A) This is one of the 15 haplotypes genealogies that showed a consistent pattern with the W-Sex fusion. The vertical lines represent the alleles of each SNP for each individual. Each vertical line represents an individual haplotype, and the alleles of all individuals are shown differentiating those of females (orange) from those of males (blue). Note that one haplotype of the females clusters with the alleles of males, while the other female allele formed a separate group (highlighted in orange). B) Position of each of the 15 SNPs in Chr9 that show genealogies consistent with a W-Sex fusion; they were not clustered in a specific region but rather distributed along the entire chromosome. The position of the SNP whose genealogy shown in A, is indicated by an arrow. (TIF) [file pgen.1011318.s032.tif]

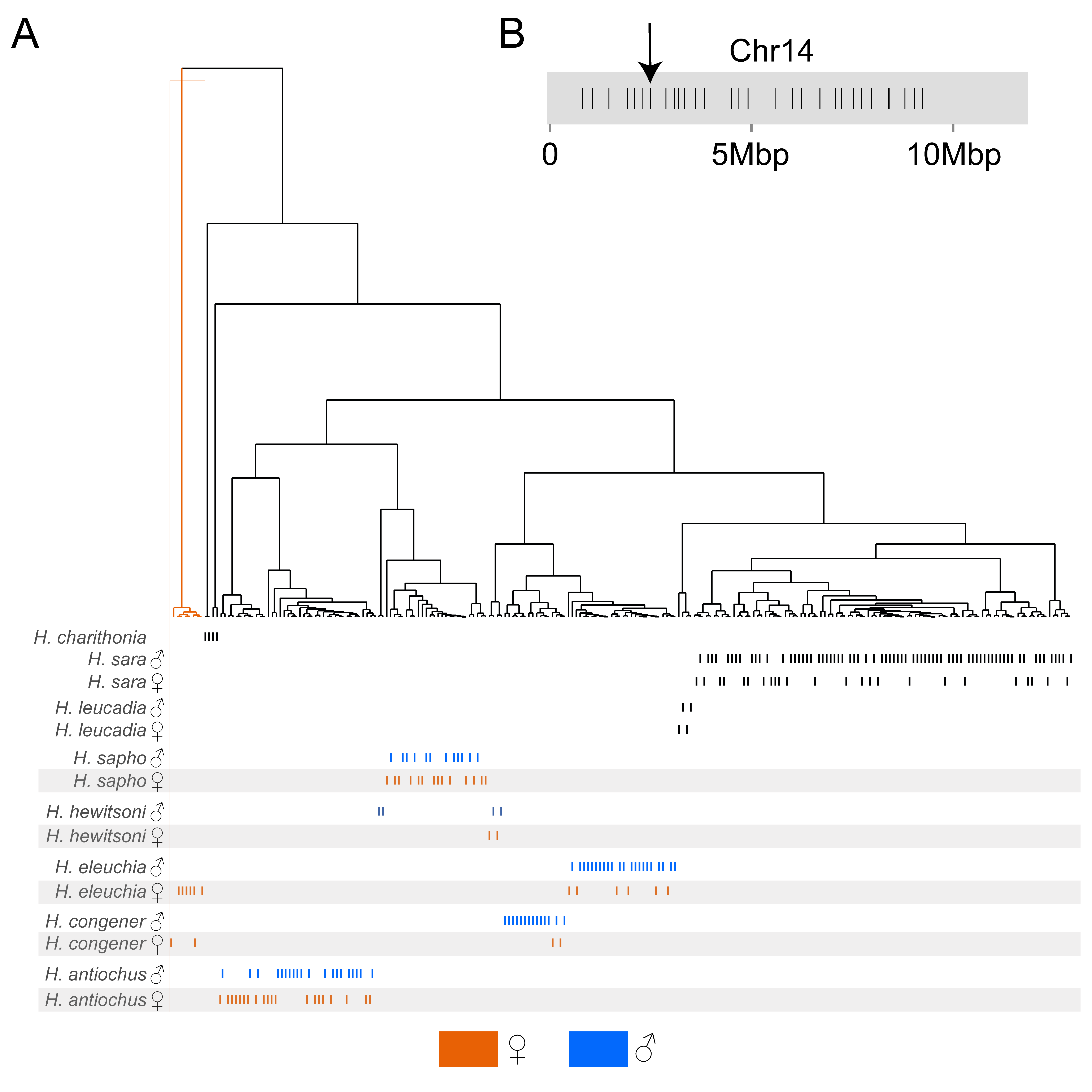

Supplement: S30 Fig — (A) This is one of the 25 haplotypes genealogies that showed a consistent pattern with the W-Sex fusion. Each vertical line represents an individual allele, and the alleles of all individuals are shown differentiating those of females (orange) from those of males (blue). Note that one allele of the females clusters with the alleles of males, while the other female allele formed a separate group (highlighted in orange). B) Position of each of the 25 SNPs in Chr14 that show genealogies consistent with a W-Sex fusion; they were not clustered in a specific region but rather distributed along the entire chromosome. The position of the SNP whose genealogy shown in A, is indicated by an arrow. (TIF) [file pgen.1011318.s033.tif]

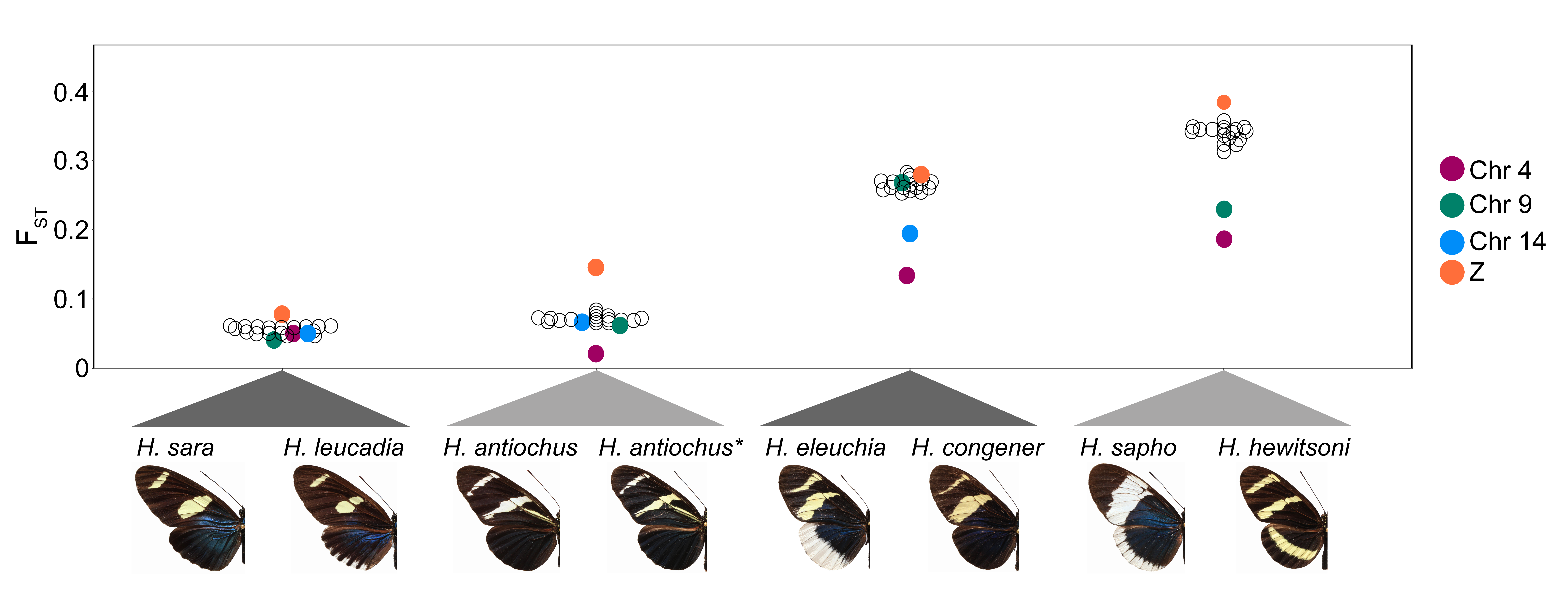

Supplement: S31 Fig — Each dot represents a chromosome, and chromosomes with evidence of Sex-A fusions are colour coded. (TIF) [file pgen.1011318.s034.tif]

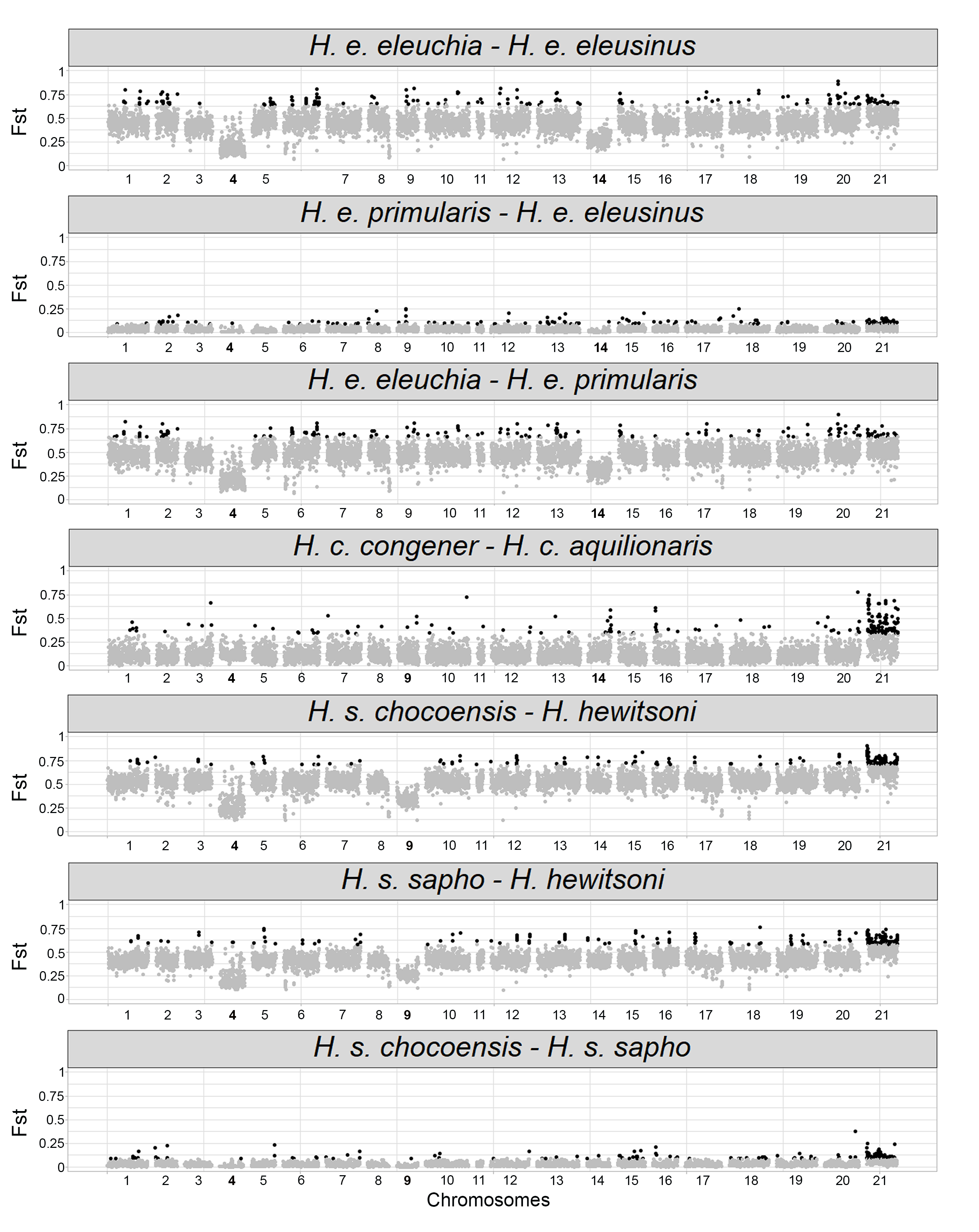

Supplement: S33 Fig — Each point represents a 50Kb window. The significance threshold is set at the top 5% of the FST values distribution tail, and black windows are those that passed this threshold. (TIF) [file pgen.1011318.s036.tif]

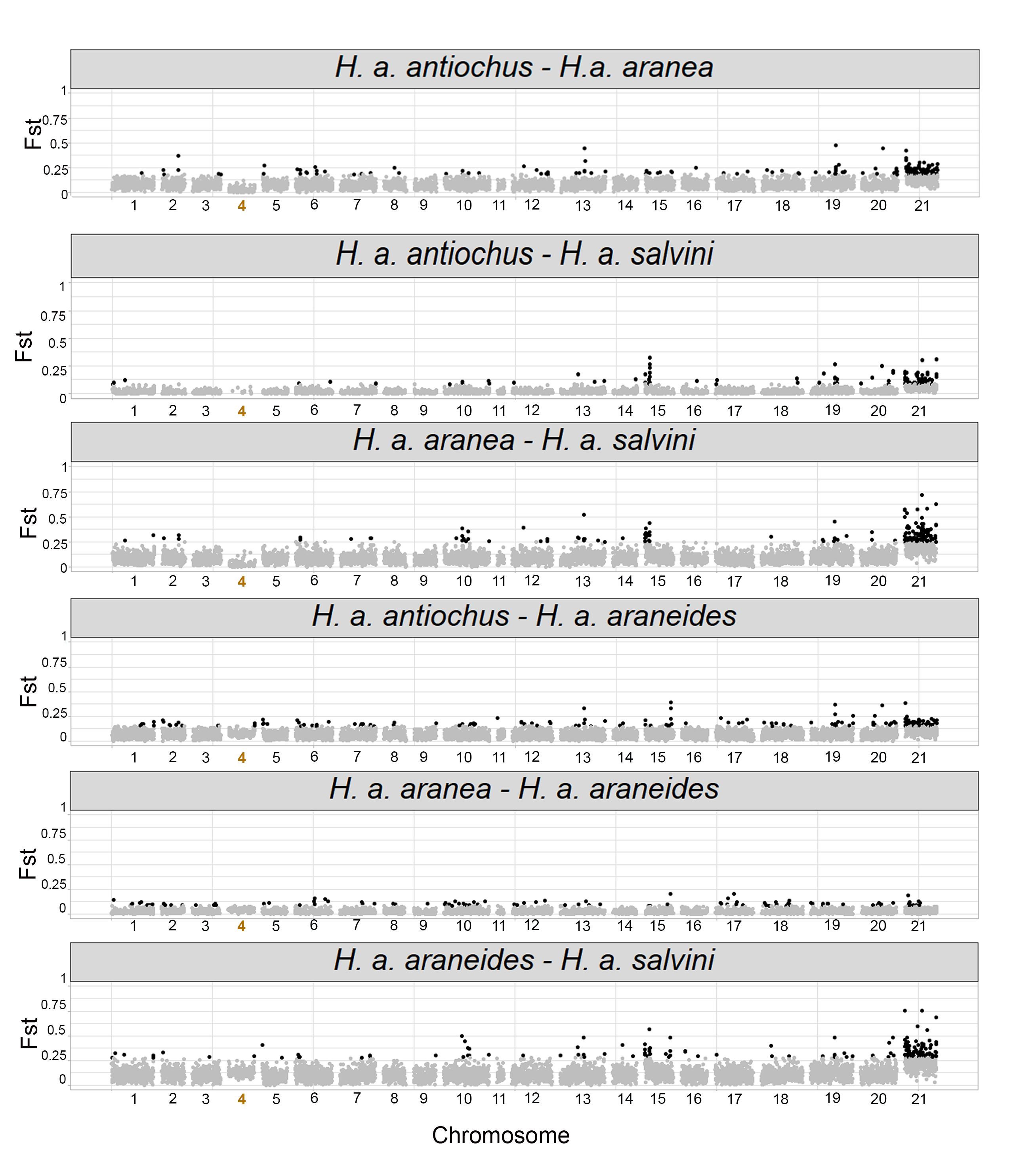

Supplement: S34 Fig — Each point represents a 50Kb window. The significance threshold is set at the top 5% of the FST values distribution tail, and black windows are those that passed this threshold. (TIF) [file pgen.1011318.s037.tif]

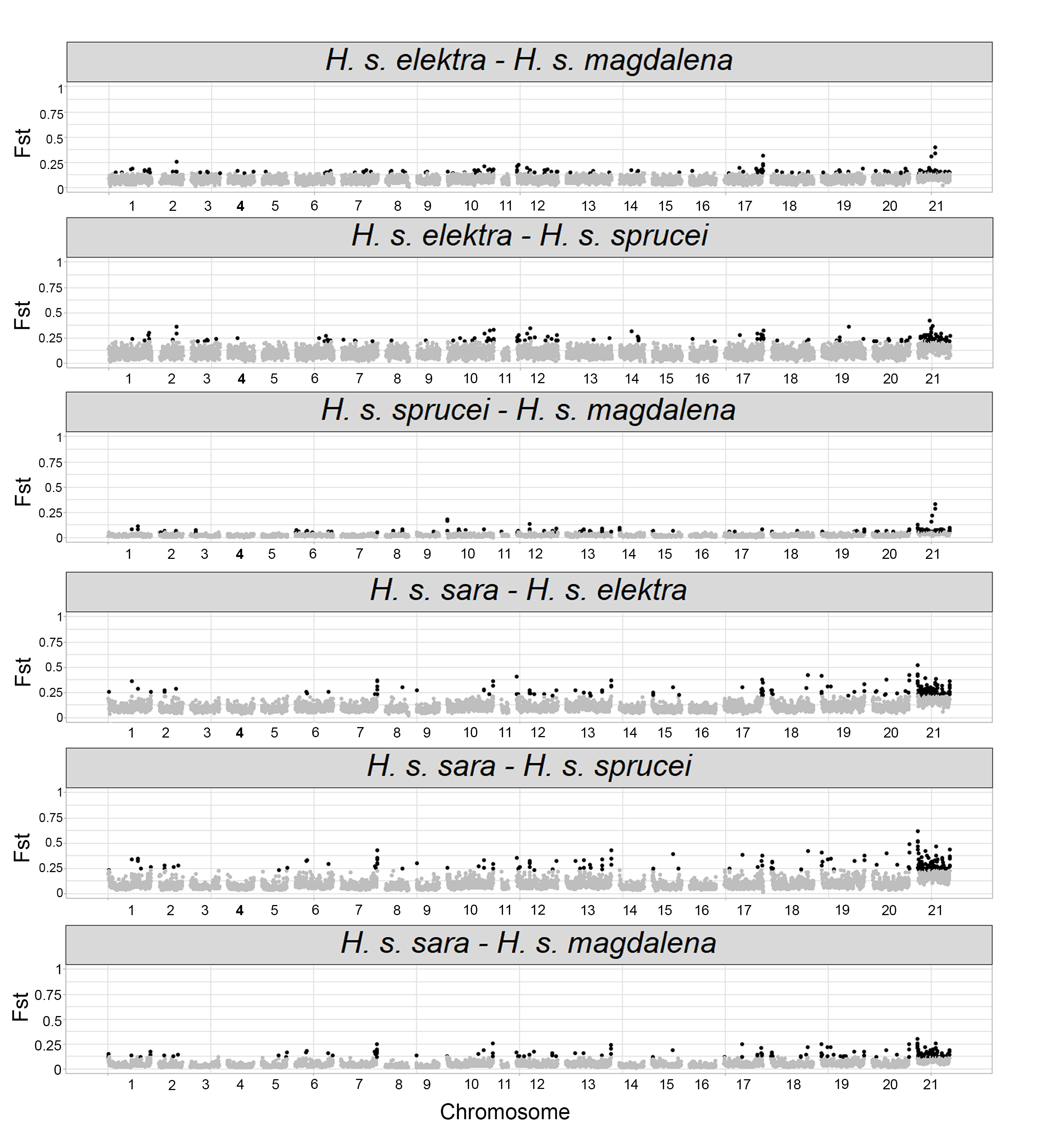

Supplement: S35 Fig — (a) FST. Each point represents a 50Kb window. The significance threshold is set at the top 5% of the FST values distribution tail, and black windows are those that passed this threshold. (TIF) [file pgen.1011318.s038.tif]

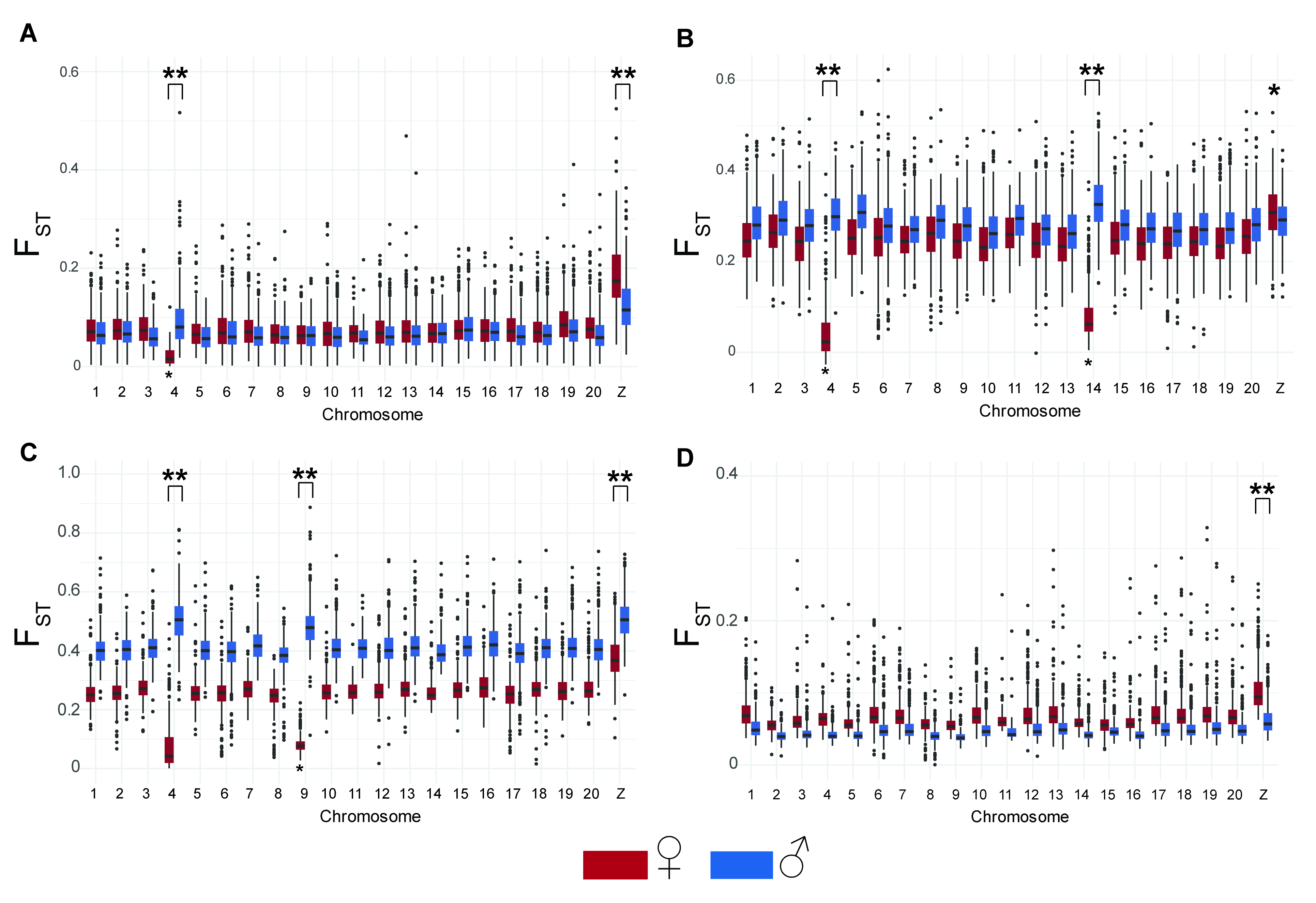

Supplement: S36 Fig — FST between (A) subspecies of H. antiochus, (B) H. congener and H. eleuchia, (C) H. sapho and H. hewitsoni, and (D) H. sara and H. leucadia. FST was calculated by sex and are colour coded. **p<0.05. (TIF) [file pgen.1011318.s039.tif]

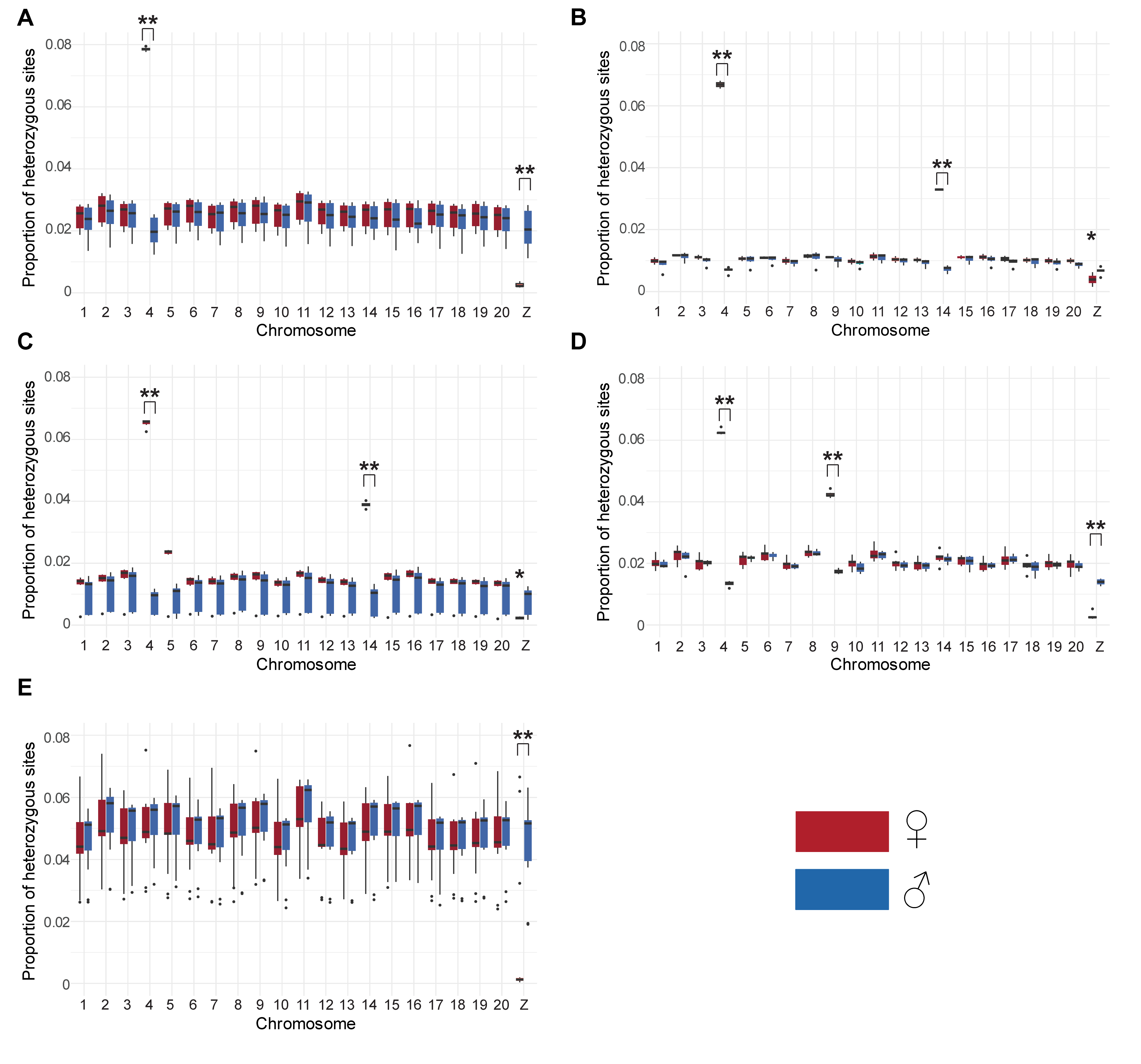

Supplement: S37 Fig — Proportion of heterozygous sites by sex in each species: (A) H. antiochus, (B) H. congener, (C) H. eleuchia, (D) H. sapho, and (E) H. sara. Sexes are colour coded. **p<0.05. (TIF) [file pgen.1011318.s040.tif]

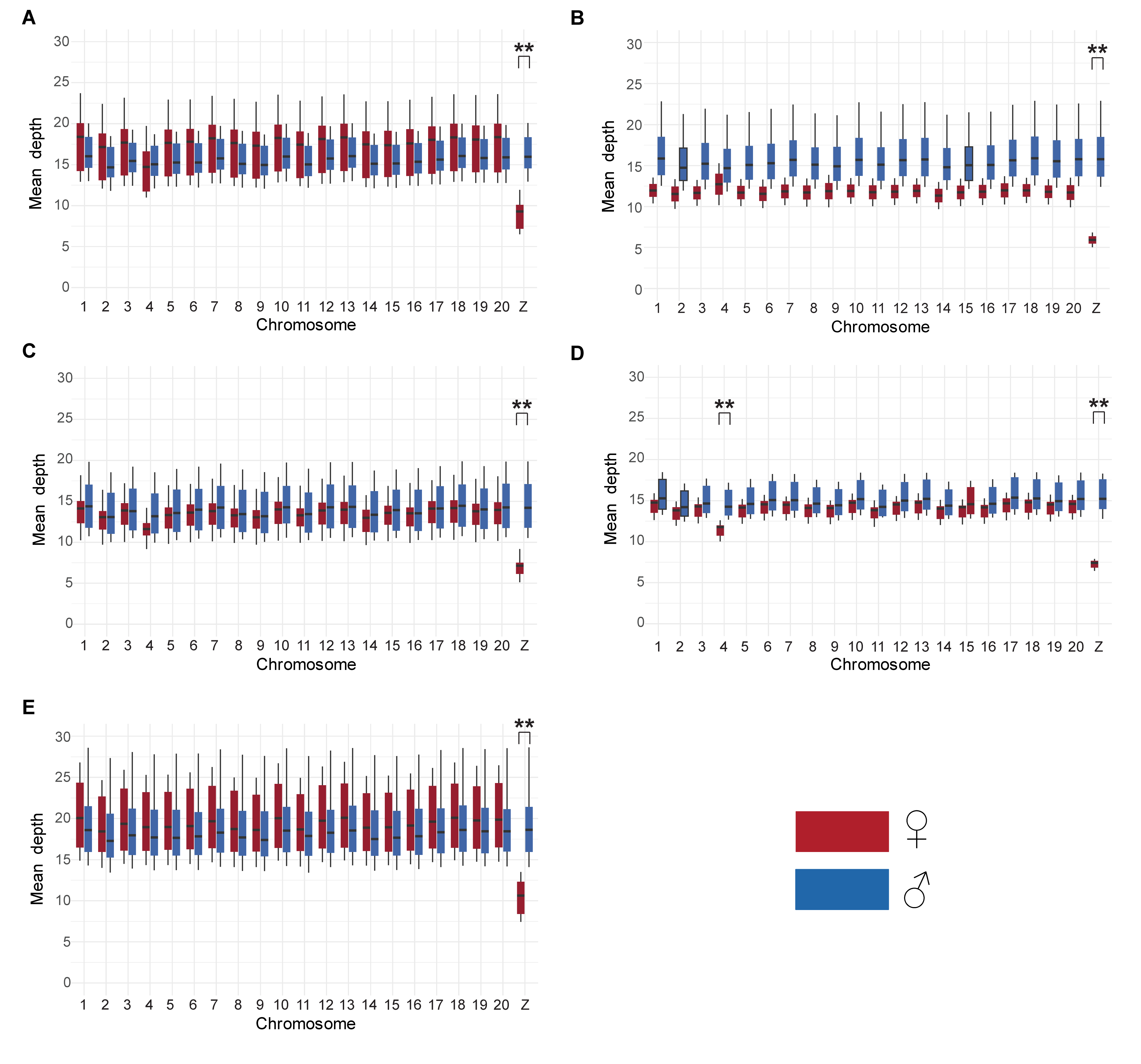

Supplement: S38 Fig — Mean depth by sex in each species: (A) H. antiochus, (B) H. congener, (C) H. eleuchia, (D) H. sapho, and (E) H. sara. Sexes are colour coded. **p<0.05. (TIF) [file pgen.1011318.s041.tif]

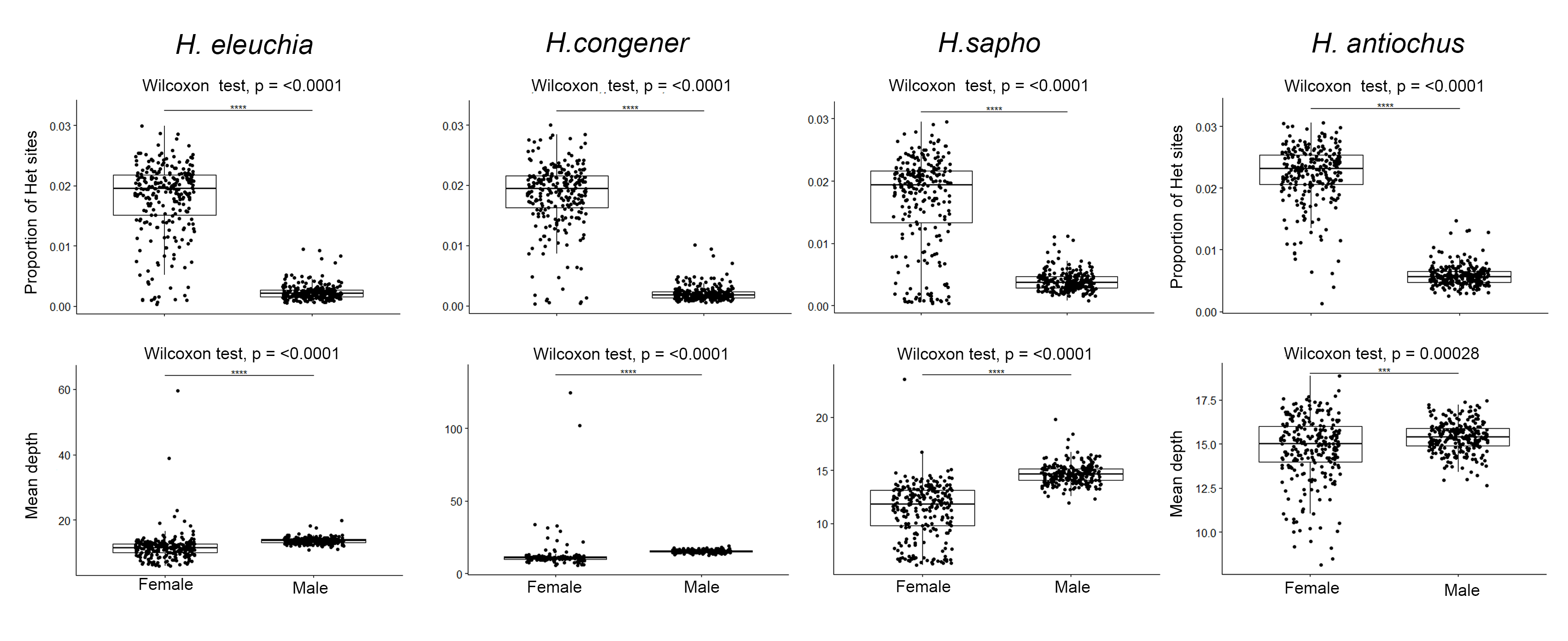

Supplement: S40 Fig — Each panel corresponds to a species with the proportion of heterozygous sites shown at the top and mean depth at the bottom. Each dot represents the average of these values across all individuals per window. ns = non-significant. (TIF) [file pgen.1011318.s043.tif]

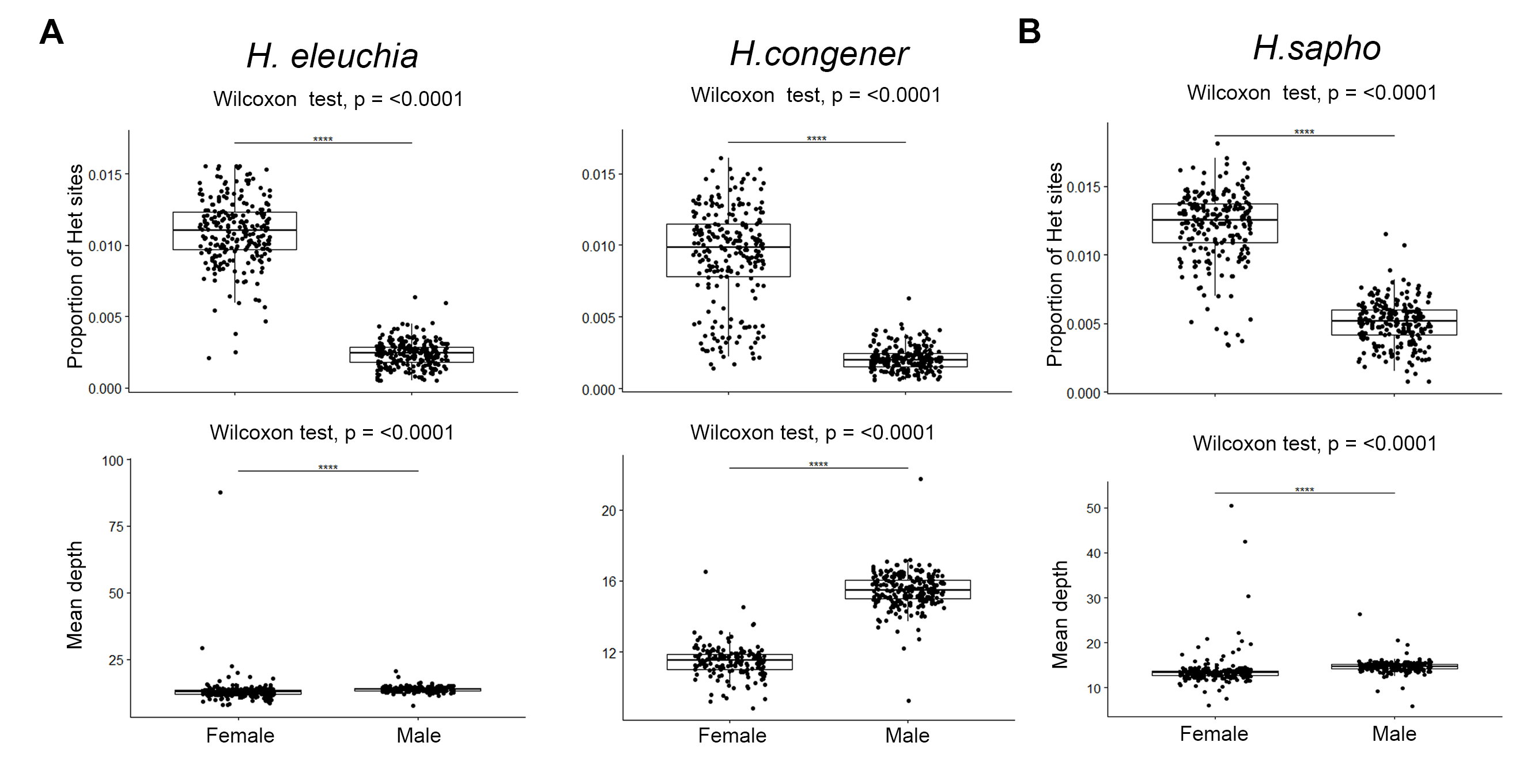

Supplement: S41 Fig — Each panel corresponds to a species with the proportion of heterozygous sites shown at the top and mean depth at the bottom. Each dot represents the average of these values across all individuals per window. ns = non-significant. (TIF) [file pgen.1011318.s044.tif]

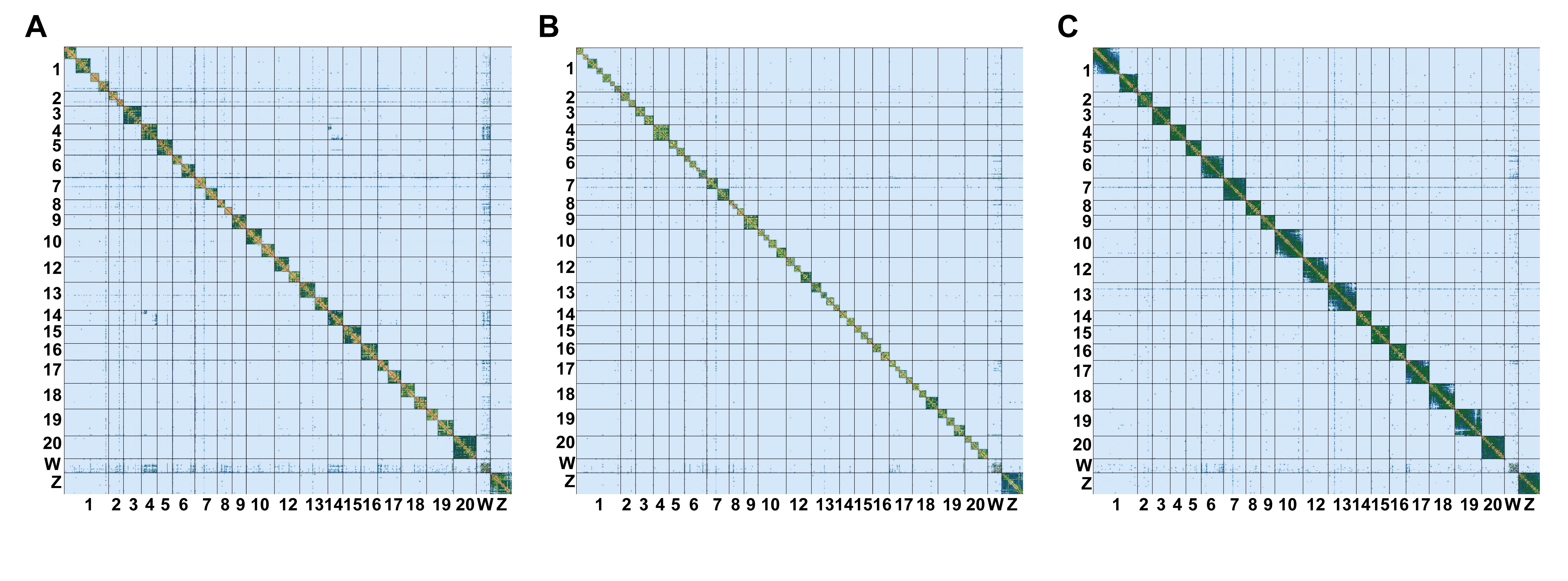

Supplement: S42 Fig — Hi-C contact heatmaps of the genome assembly of H. charithonia against: (A) the H. sara female (Hi-C used to assemble the genome reported here), (B) the H. sapho male, and (C) the female of H. congener (TIF) [file pgen.1011318.s045.tif]
