# Supplementary material for: Genomic evidence reveals three W-autosome fusions in Heliconius butterflies
Source: PLoS Genet. 2024 Jul 18;20(7):e1011318. doi: 10.1371/journal.pgen.1011318 (PMC11257349; doi:10.1371/journal.pgen.1011318)
Supplement: Attachment — Submitted filename: Response to Reviewers_Rueda-M_N., et_al_2024.pdf [file pgen.1011318.s046.pdf]

Responses to the comments by the guest editor

This is an interesting paper concerning the genomics of a phylogenetic sub-group of Heliconius butterflies that has hitherto not been tackled. I have good evidence that the clade was in fact deliberately ignored for genome sequencing to avoid assembly and interpretation complications due to the known rampant chromosomal evolution previously demonstrated by testes-based chromosomal counts. Almost all Heliconius are highly syntenic and have n = 21 chromosomes, but the sara-sapho group may have n = 21-60, and are sometimes apparently polymorphic for chromosome number within species. And the expectation of interpretation difficulties has now been amply demonstrated by the current authors! The current work provides the first genomic analysis of that clade, and is based on a high quality chromosomal scale reference genome for H. sara (n = 21) as well as reasonably high coverage Illumina resequence data for all the other species in the clade and many of their subspecies.

Reviewer 1 recommends rejection; Reviewer 2 recommends minor revision, and Reviewer 3 major revision. I am therefore inclined to suggest major revision, but I do think that the very important critiques of Reviewer 1 need to be answered. My own feelings about the manuscript were disappointment that the paper was not more definitive. The authors have some strong evidence for genomic shenanigans, but I agree with the reviewers that they have only indirect evidence to support their claims about fusion of autosomes with sex-chromosomes in the sapho-eleuchia subclade. This is because no assemblies were done of the putatively fused species, and so the “phylogenetic” pattern of males and females appearing on separate branches, strange heterozygosity differences in females, and patterns of divergence are the only evidence of sex-autosome chromosomal fusions.

Answer: We thank the guest editor for the feedback and giving us a chance to resubmit. We have now resolved the lack of a definitive answer with new Hi-C data that strongly supports that the fusions are with the W chromosome. We now also include haplotype-based phylogenies that clearly show that the W-autosome fusions are ancestral to five species (Chr4 fusion), or two species each (Chr9/14 fusions). We have also addressed all other comments and hope that they are found to be satisfactory.

1. The reviewers do not dwell on this, but I was rather shocked that there is not more discussion of the high quality reference genome of H. sara. Instead, all we get is a 6-line paragraph with the title “Genome assembly” in the results. I infer that the genome assembled is H. sara, but the species is not even mentioned in the Results! There are no genome coverage, or other statistics that I could find (except some mention of BUSCO), and no mention of the sequencing of the W chromosome. If it was a female sequenced (maybe mention in the results, as well as in methods!) there should be a W chromosome, if there is one. But the results just state “36 scaffolds to 20 chromosomes and the Z chromosome”. If there is a W, then that should appear as a 22nd chromosome with 50% coverage compared to autosomes. Does it have any genes on it? And the Z should also have 50% coverage compared to autosomes.

As it happens, and probably unknown to the authors because of recent pre-publication, a H. charithonia genome has also been sequenced using long-read data, and shows that the W chromosome contains a female-specific UV receptor gene (Chakraborty, M., Lara, A.G., Dang, A., McCulloch, K.J., Rainbow, D., Carter, D., Ngo, L.T., Solares, E., Said, I., Corbett-Detig, R., Gilbert, L.E., Emerson, J.J., & Briscoe, A.D. 2022. Sex-linked gene traffic underlies the acquisition of sexually dimorphic UV color vision in Heliconius butterflies. bioRxiv:2022.2007.2004.498748). So I'd be interested to know: did the authors find the same W chromosome in H. sara, which is reasonably closely related to H. charithonia (assuming the Briscoe lab are prepared to share the genome sequence)? Prior to the Chakraborty et al. paper, no W chromosome had ever been identified in the genus Heliconius. In view of the suggestion by the current authors that the fusions could be Z – A or W – A, it does seem important to mention whether they found a W chromosome at all in the group! Given the assembly of the W in charithonia, are there any signs of a possibly homologous W chromosome in all the other species studied here in addition to the autosomal fusion parts?

Answer: We thank the editor for pointing out this omission and fully agree that more information on the reference genome is required. We now provide detailed information on the genome assembly in the methods and quality metrics in the results. We have added the species and sex of the reference genome to the main document in lines 118-119. Additionally, we included information on the specimens used in Supplementary Table S1. Statistics related to the assembly have been incorporated into the Methods in the section "Genome assembly of *Heliconius sara*". The reference individual is indeed female but as the PacBio data stems from a time before HiFi reads existed (CLR data), the W could not be assembled. However, we have now identified W scaffolds by comparing the sequencing coverage of *H. sara* females and males in the scaffolds that had previously not been assigned to a chromosome (Please see the section "Genome assembly of *Heliconius sara*" in lines 514). These W scaffolds are syntenic with the W of *H. charithonia* (see Fig S1). Interestingly, females of the species with the sex-autosome fusions from the *sapho* subclade (*H. antiochus*, *H. sapho*, *H. hewitsoni*, *H. eleuchia*, and *H. congener*) do not map to these W scaffolds, indicating that the W of these species diverged too much from the *H. sara* W or that they lack the ancestral W (For more details, see results in lines 137-148 and Fig S2).

2. I also was disappointed that the manuscript seems poorly written and in places is not clear or grammatical. I am aware that the main authors are not native English speakers and this is therefore understandable. It is also important, I think, that the Colombian authors, in whose country the vast majority of the species occur, should be the ones writing this paper. However, some of the authors are native speakers, and one has recently joined the "Royal Society" and so should be able to speak the King's English! It seems to me that they ought to be able to help out more than they appear to have done.

Answer: We have revised the manuscript for clarity and grammar.

3. Reviewer 1 suggests that in addition to Z-A or W-A fusion, we may actually have both, and that chromosome squashes and counting in both males and females should be done to check. I imagine this is not feasible with Heliconius currently; most chromosome counts have been done only with males. Instead, I would favour a genetics-based approach involving sequencing the brood of offspring of a single female instead, or a new set of long-read assemblies. But these would both be a lot more work, including new field work. I suggest the authors instead say what they have found and what it means, but mine their existing data better in support of their interesting hypotheses.

Answer: Thanks to the deadline extension, we have now managed to catch new butterflies and generate Hi-C data for a female *H. congener* (Sex-A fusion with 4+14) and a male *H. sapho* (Sex-A fusion with 4+9). We mapped the new Hi-C data against our *H. sara* reference genome. This new experimental design allowed us to conclude that the fusions are all with the W, as the male *H. sapho* shows no fusion and the female *H. congener* is heterozygous for the chr4-14 fusion. For more details, please refer to the manuscript in section “Evidence for three W-autosome fusions”.

4. I’m not a chromosomal expert (although Reviewer 1 is), but I would have thought that the two or three possible hypotheses depend on sex determination in Heliconius, and we do not understand that yet. If the W chromosome contains a gene that determines femaleness, as in Bombyx, then yes, the W-A fusion might create a second Z chromosome from the unfused autosome, so that you have Z1 Z2(the former A) and W-A where the W-A becomes a neo-W. If on the other hand, it is the dosage of the Z chromosome is important in sex-determination, as in Drosophila, a W-A fusion could instead lead to the loss of the W chromosome and the fused part could form a new part of the autosome, once fixed. And of course, we don’t even know whether these putative fusions are fixed.

Answer: We do indeed not know if the sex determination locus in *Heliconius* butterflies is located on the Z or W. However, our findings of W-fusions mean that by definition the W-4-9/14 is a neo-W and the corresponding chromosomes 4 and 9/14 thus become Z chromosomes by definition. We now use these terms more explicitly. Interestingly, we find that none of the females in the *sapho* subclade have reads mapping to the W scaffolds of *H. sara*. This may just be due to the fast divergence of the W generally seen in Lepidoptera or may indeed suggest that the original W has degraded completely in the *sapho* subclade species. This would mean that the sex identifier is likely on the Z. Given that many Lepidoptera species show high turnover of W chromosomes or have lost it altogether (Chen et al., 2023), it is likely that the Z chromosome is sex-determining in most Lepidoptera. Identifying the mechanism of sex determination and understanding how dosage compensation works in *Heliconius* (Walters, et al., 2015 and Catalan, et al., 2018) are important issues that continue to be an enigma in this genus. However, these questions go beyond the scope of this study.

5. Here are some critiques I have of the main results: In Fig. 1 you have found a ML tree for the whole genome, but you make no attempt to accommodate the

mixed signal from different loci. I suggest you try to adopt a species tree approach to obtain the species phylogeny, perhaps for males only (to avoid female-specific neo-W chromosomes). As there is no evidence for current gene flow in this group, even an analysis based on the assumption of split without gene flow would be better than this concatenated analysis (which implicitly assumes a lack of recombination at all). A firm hypothesis for the species tree would serve a useful purpose for understanding the comparative analysis of the chromosomal fusions, e.g. in Fig. 6, and for the chromosome-specific contatenated ML trees.

Answer: We thank the guest editor for this suggestion. We have now added an Astral species tree using two male individuals from each subspecies (to avoid biases caused by the shared sex-autosome fusion). The resulting topology was consistent with the whole genome tree. The methods and results have been added in lines 598-609 and 179-185, respectively.

6. The “phylogenetic”, PCA, Fst and Dxy, whole chromosome heterozygosity, and coverage analyses of males and females in Figs. 2,3,4,5 are all somewhat confusing because the two chromosome morphs in the autosomal fusion chromosomes are presumably lumped together, to give a mixed signal. In Fig. 3 the “windows clustering by sex” also cluster somewhat by species; and in the “windows clustering by species” there is still some clustering by sex evident. Thus the “grey” and “black” areas in Fig. 3. C are not completely successful in partitioning the sex and species clustering; again you appear to have mixed signal in both grey and black regions of chromosome 4.

Answer: The editor is correct in assuming that the mixed signals observed in the phylogenetic trees and PCA results are due to “lumping together chromosome morphs”. We conducted these analyses without separating the haplotypes and females thus appear as intermediate. To separate the haplotypes, we have now phased the data with whatsHap and shapeit4 from the population data in this study. We then run Relate to obtain SNP genealogies (For more details, refer to section “Haplotype-based phylogenetic analysis on chromosomes 4, 9 and 14”). Additionally, we separated the haplotypes from the HiC data using a “Chomper” from Mackintosh et al., 2022 (Please see section “Evidence for three W-autosome fusions”). Both analyses confirmed the presence of one fused haplotype and the other unfused in females but not in males (Please see figures 5 and 7). Furthermore, the haplotypes from the HiC data indicated that this chromosomal rearrangement is more complex than just simple fusions, suggesting the presence of additional translocations or inversions. We have removed the PCA and Dxy analyses and have added F_{ST} analyses for females and males separately, which shows that the drop in F_{ST} on the chromosomes fused with the W is only seen in females.

7. In Fig. 4, I assume you use the Hudson & Maddison definition Fst = 1 – (piW/piB), so Fst will be higher if piB (i.e. Dxy) is higher, and lower if piW (i.e. heterozygosity) is higher. So it is a little unclear what Fig. 4 means. The Fst results would be an artefact of the changes in Dxy and/or piW – see Cruickshank & Hahn.

Answer: The editor is correct. We used the Hudson & Maddison equation Fst = 1 – (piW/piB). Therefore, the low Fst on the chromosomes fused with the W is indeed an artifact of high piW within each population due to high divergence between the fused and unfused haplotypes in each population. Our Relate analyses additionally show that the W-A fusions are ancestral to the sister species compared and females of one species thus share a haplotype with the females of the other species, reducing the differentiation between the species. So piB is not increased by the same extent. We have added a clearer explanation of what a low Fst means in lines (283-286 and 303-304). We decided to keep this analysis because it allows us to demonstrate how SA fusions look in WGS datasets. We have now also added Fst plots for females and males, separately, which shows that the drop in Fst is only seen in females (Please see Figure 4).

8. In Figs 5 and 6 I am unconvinced by the heterozygosity and coverage analyses, and want to know more. For example, I'd expect the coverage to be halved in the female when the Z chromosome is used in the females; the "standardized" heterozygosity and mean depth measures don't seem to show this, though I don't really know what they do mean (it's not explained in the figure legend). Also, given that some parts of the fused autosomes in the female appear rather similar in male and female, while most other parts appear divergent between the sexes (high heterozygosity and lowered coverage in females) (Fig. 7), the whole-chromosome heterozygosity and coverage measures in Figs. 5 and 6 seem very poor indicators of what is going on. Also in Fig. 7, in order to accommodate the very high coverage in apparent repeat regions of the female, the scale of mean depth is so small that one can't distinguish what is going on, with the approx. average 15x coverage, between males and females; in H. sara, the overlap seems complete (as perhaps expected), but only the blue male lines are visible in the figure. Can the scale be increased, with repeat regions annotated but not shown on the scale?

Answer: The figure of heterozygosity and mean depth by chromosome has been modified for clarity (Please see Figure 5). We also have added a more detailed explanation in the legend of what they represent. The new figures clearly show that the chromosomes involved in the fusion (4, 9, and 14) have a high number of sites where females are heterozygous, as a result of the presence of two highly divergent haplotypes. This figure also shows that these chromosomes exhibit a lower mean depth than the other chromosomes in females, caused by reduced mapping of the fused haplotype to the reference genome. As the editor suggests, the Z chromosome in females shows a lower sequence coverage than that of males and half compared to autosomal chromosomes. In Figure 6, we have adjusted the scale of the Y-axis to better appreciate the differences in heterozygosity and mean depth between the sexes. Here, it becomes clear that some regions show reduced sequencing depth and near zero heterozygosity, indicating that these are regions where the fused haplotype diverged too much from the reference to be mappable.

9. It seems to me that the problem with all these mixed signals is that one gets very little feel for the actual haplotypes underlying these signals. But Illumina data at 15x and higher allows for reasonably accurate heterozygous genotype calling;

and given that LD would presumably be very high along the new W version of the autosomal fusion that has no recombination (see reviewer 2's comment) it seems likely that the differences could be phased for each individual female when you have one or more female and one or more male sequences. See also the comments from reviewer 2.

Answer: Sadly, phasing across the entire chromosome does not work for a phylogenetic dataset like this, particularly for the species with only one (*H. hewitsoni*) or two females (*H. congener*). However, we managed to get decent short-range phasing with a combination of read-based phasing/haplotype assembly and statistical phasing. This allowed us to generate marginal phylogenies in Relate that show the haplotype structure well and allow readers to get a better feel for these haplotypes (see also our next answer). In addition, the Hi-C data provides a robust confirmation of the W-fusion in females and that it is present in heterozygous state in females only.

10. Thinking about this a little more, it seems to me that providing you can call high confidence heterozygous sites in females, and the SNP is usually homozygous in males (due to the much lower heterozygosity in males as shown in Fig. 7), then one should usually be able to call the female-specific base and the male-specific base in females. For instance, in chromosome 4, if we call these neo Z and neo W haplotypes 4Z and 4W, then for many regions you'd know the sequence for each haplotype. You'd expect the 4W chromosome not to recombine, so it should be fairly easy to pull out the 4W from each female and test for LD decay along the chromosome compared with the 4Z neo-chromosome (can do this with as few as 4 individual diploid sequences, see Heliconius Genome Consortium 2012 supplement). This might be messed up in some SNPs, or in reads with no good SNPs, or in reads with polymorphisms in 4Z as well. But you wouldn't expect to find any of the divergent 4W haplotypes in males at all, so you should be able to separate a lot of the chromosome's reads. It might even be possible to use some graph-based assembly program for Illumina (e.g. like DISCOVAR) to at least get slightly longer haplospecific contigs than just the raw reads. However, even the individual SNPs on individual aligned reads should be possible to phase in this way by divergence from the males. Standard methods of LD-based phasing might not work very well, but in principle, I guess that simple-minded divergence based 4W SNPs should work right along the chromosome. You could then construct haploid trees of the 4Z and the 4W chromosomes and separate a large portion of the haploid reads in each individual female. Then all the mixed signals would go away, and the paper would be on firmer ground. You are still unlikely to be able to find the fusion points, but at least you'd have bioinformatic evidence of different 4W haplotypes that show similarity among females, but less similarity between 4W in females and 4Z in males. This would "explain" the heterozygosity results, for example.

Answer: Thank you for the suggestion. To remove the mixed signals, we decided to phase our data combining two methods: WhatsHap, which is a haplotype

assembly technique using the phase information from read pairs (Patterson et al., 2015), and SHAPEIT4, a statistical phasing method using information across all individuals (Delaneau & Marchini, 2014). We then inferred SNP coalescent genealogies from the phased data across each chromosome using Relate. Because any possible Sex-A fusion would produce heterozygous females and homozygous males, we identified and selected sites with this pattern to visualize their genealogies (Please see section: "Haplotype-based phylogenetic analysis on chromosomes 4, 9 and 14"). As mentioned before, we also separated the haplotypes from the HiC data using "Chomper" from Mackintosh et al., 2022 (Please see section "Evidence for three W-autosome fusions"). Both analyses confirmed the presence of one fused and one unfused haplotype in females but not in males (Please see figures 3 and 7).

11. On another point, I'd like to see all the precise geographical localities and if possible GPS coordinates for each specimen; the country is nice (mostly Colombia), but the precise localities should be listed. In summary, I again would like to apologise for the many delays in processing this manuscript, but I hope you do understand that it was tricky to understand and come to a decision. I hope that you find these comments useful, and feel free to ignore any that are impossible! (You should say so if that's the case). You should also pay attention to all three reviewers' comments, and answer them in a detailed reply. I personally really look forward to seeing an improved version.

Answer: Table S1 contains this information. We have now also added the reference genome individuals to that table. Many thanks for your thoughtful comments. We think that they have massively improved our manuscript.

Responses to Reviewer #1:

Reviewer #1: In this manuscript, the authors present the sequencing of seven species of Heliconius butterflies (as well as some subspecies) and use some bioinformatic analyses to examine genome-wide phylogenetic relationships, the degree of genomic differentiation among species, and the evolution of the sex chromosome. In particular, the authors address the possibility that fusions of the sex chromosome and autosome have occurred in some clades of Heliconius butterflies.

Overall, some of the data presented here may helpful in understanding molecular phylogeny and the degree of genomic differentiation in species of the genus Heliconius. However, I have major concerns about the analysis and/or presentation of sex chromosome evolution (see below).

1. In L234-L244, L306, L318-L320 and Fig. 5, the authors give two possibilities for neo-sex chromosomes, either Z-A or W-A fusion. However, in addition to the two scenarios mentioned by the authors, I think a scenario in which both W and Z fuse with autosome(s), resulting in a neo-Wneo-Z sex chromosome constitution, is also possible. Also in this case, some mutations may occur in the ancestral autosomal part of the neo-W chromosomes, leading to sex-specific differences in heterozygosity. I think that chromosome counting of both sexes enable to verify which SA fusion (Z-A or W-A or both of Z-A and W-A) has occurred in each Heliconius species.
   (1) Both sexes have the same number of chromosomes = both W and Z fuse with the autosome (sex chromosome constitution is neo-Wneo-Z)
   (2) Males have one more chromosome than females = W-A fusion (sex chromosome constitution is neo-WZ1Z2 shown in Fig. 5B, Z2 is used to be autosome)
   (3) Females have one more chromosome than males = Z-A fusion (sex chromosome constitution is neo-ZW1W2 shown in Fig. 5A, W2 is used to be autosome)

Answer: Thank you for the suggestion. We fully agree that W and Z fusions could have both occurred and now make this clearer in the text (Lines 225-236, Fig. 2 legend). We have now generated Hi-C data for *H. congener* and *H. sapho* and mapped them against our reference genome of *H. sara* and to the *H. charithonia* genome. We found that the fusion is only present in females and is not with the Z chromosome. Therefore, we conclude that all fusions are likely with the W chromosome. For more details, please refer to the manuscript in section “Evidence for three W-autosome fusions”.

2. The authors have presented the chromosome number of each species in Fig. 1. According to the article (ref no. 36, Brown et al 1992 Hereditas), the chromosome numbers seem to be for males. Why do not the authors show the chromosome numbers of both sexes? Clarifying numbers of chromosome in both sexes can verify which SA fusion occurred in each species, as I stated in above. I think that the information about chromosome numbers in both sexes is important to fully demonstrate sex chromosome evolution that

accompanies the SA fusion. This should be clarified in this manuscript. Of course, genome assemblies of each species also help to verify which the SA fusion (Z-A or W-A or both of Z-A and W-A) occurred in each Heliconius species. Why did the authors only assemble the genome of H. sara? Why did the authors not assemble the genomes of other Heliconius species? If the authors want to fully demonstrate the neo-sex chromosome evolution, the combination of some molecular cytogenetic works (especially, chromosome counting in both sexes) and the genome assembly should be a more appropriate analysis.

Answer: Thank you for the suggestions. It is true that clearly establishing the number of chromosomes in species and sexes would provide greater clarity on these chromosomal rearrangements. We have now added Hi-C data of two individuals, which clearly confirms the high number of chromosomes in *H. congener* and *H. sapho* and reveals that the fusions are with the W and not with the Z. We hope to obtain reference genomes for all *sapho* subclade species in the future to confirm these chromosomes numbers and continue with this interesting study, but this is outside the scope of this study. Finally, we would like to emphasize that while it is becoming more feasible to generate reference genomes, the high repeat contents of W and Y chromosomes makes their assembly challenging and thus they are often missing in reference genomes and fusions may thus be missed if only reference genomes are used. Our study highlights that short-read population WGS data including both sexes can be very valuable to detect SA fusions. We show what patterns to expect if the fusions are recent enough that the previously autosomal chromosomes are still diploid in both sexes.

3. The authors seem to identify female-specific haplotypes in some Heliconius species. From the results of female-specific haplotypes, in Fig. 5 or in the discussion, the authors suggest the possibility of mutations and/or structural variants on W or neo-W chromosomes. Why did the authors not characterize the mutations and/or structural variants, which could be the accumulation of repetitive sequences and/or gene duplications, etc., in the female-specific sequences? As the authors stated in the discussion, lepidopteran females have no recombination, resulting in the W chromosome degenerations by highly accumulation of repetitive sequences or gene duplications, etc., on the W (or neo-W) chromosomes. Verification that repetitive sequences or gene duplications, etc., have accumulated in the female-specific sequences will provide clear evidence not only for SA fusions, but also for W (or neo-W) chromosome degenerations accompanied by SA fusions. This is likely to be important to fully demonstrate the evolution of sex chromosomes in Heliconius butterflies.

Answer: Thank you for the suggestion. We fully agree that we would expect the accumulation of repeats, TE expansions, gene duplications etc on the female specific haplotypes. However, these would be missing in the reference genome and by mapping short-read data to the *H. sara* genome, we would miss most of them. However, some repeat expansions or duplications are expected to be visible as peaks of high sequencing depth and we do indeed see this.

Based on the above comments, I am afraid that I have to come to the conclusion that the authors have not fully demonstrated the evolution of neo-sex chromosomes in the sapho clade of Heliconius butterflies. As far as I know, many studies in several lepidopteran species using molecular cytogenetic analysis and/or genome assembly have clearly shown that neo-sex chromosomes have arisen from the fusion of the sex chromosome and autosome, and fully demonstrate the neo-sex chromosome evolution. Compared with their reports, the results on sex chromosome evolution in this manuscript seem to be weak and incomplete, and although several bioinformatics analyses have been well performed in this manuscript, only limited information is provided for understanding sex chromosome evolution and evolutionary genomics in Lepidoptera. Therefore, the results shown in this manuscript are not a sufficient advance, they lack general interest, and in my opinion this work seems more suitable for a more specific journal.

Other comments:

4. L37-L38, “21 chromosomes” or “60 chromosomes”: It would be better to indicate that these are haploid numbers.

Answer: Agreed, we have now done that.

5. L102, “21 chromosomes”: Is chromosome 21 Z chromosome in Heliconius butterflies? If so, it would be better to provide the information that chromosome 21 of Heliconius butterflies is a Z chromosome.

Answer: Thanks, we have now done that.

6. L109-L110, “seven species in the sara/sapho”: The chromosome numbers were shown in Fig. 1. However, I strongly suggest that the authors provide the chromosome numbers (karyotypes) of both sexes and the sex chromosome constitutions in both sexes of these seven species (see my major comment).

Answer: Thank you for the suggestion. However, this is not possible since we do not have information on either the number of chromosomes per sex or the sex chromosome constitutions by species. Additionally, in the 1992 Hereditas article by Brown et al., it is not clear whether all individuals are males or if both sexes were studied. The only suggestion for some subspecies is that the number varies among the sampled individuals. It is true that clearly establishing the number of chromosomes in species and sexes would provide greater clarity on these chromosomal rearrangements. We hope to obtain reference genomes for all species in the *sapho* subclade in the future to confirm these chromosome numbers and continue with this interesting study, but this is beyond the scope of the current research.

7. L118, “final assembled genome”: Is this the final assembled genome of Heliconius sara? The authors seem to state this information in Materials and Methods, but it would be better to indicate the information here as well.

Answer: Thanks, we have clarified it.

8. L214-L227: I can see that the authors are somehow trying to connect the results of Fst between species (or populations) with the conclusion of sex chromosome evolution stated in the discussion. However, I could not understand why the authors show the results of absolute divergence (x), because the conclusion that follows from the results of Dxy values between species (or populations) is missing in this paragraph or discussion. What kind of information about sex chromosome evolution or the degree of genomic differentiation do the absolute divergence (Dxy) results provide?

Answer: We agree with the reviewer. We have removed the Dxy results and instead added information about the Fst by sexes. This permitted us to show that the reduced F_{ST} on certain chromosomes is only in females and not in males. We have adjusted the discussion on F_{ST}.

9. L217-L218, “absolute divergence (DXY) was highest in H. sara vs. H. leucadia”: It looks that the value of absolute divergence (DXY) in H. sara vs. H. leucadia was lowest. The authors mean that the value in “H. sapho vs. H. hewitsoni” was highest?

Answer: As mentioned earlier, we have removed the Dxy information.

10. L218-L219, “all other species pairs exhibited a similar degree of divergence”: It looks not similar in H. antiochus (Amazonian) vs H. antiochus (Andean), H. congener vs H. eleuchia, H. sapho vs. H. hewitsoni.

Answer: As mentioned earlier, we have removed the Dxy information.

11. L236, “females of Heliconius are ZW and males are ZZ”: Which Heliconius species did the authors mean? How did the authors know this? The authors should cite some article in this sentence.

Answer: Most Lepidotpera are ZW/ZZ. Since our last submission a new genome of *Heliconius charithonia* with assembled that confirmed the ZW chromosomes and we have also managed to identify some W scaffolds in our *H. sara* genome and now show that the Z between this genome is fully syntenic with the Z chromosomes in other *Heliconius* genomes. We thus think that the assumption that all *Heliconius* are ZW/ZZ is justified.

12. L301-L304, “This results…….reduced FST pattern”: This sentence is unclear. Do “populations” in this sentence mean species?

Answer: We clarified this point in lines 283-286 and 303-304.

13. L361, “SAMEA5394385”: Is this accession No? If this is accession No, please put this number in the column (Accession) of Table S1. If not, please indicate accession No. of assembled genome in H. sara.

Answer: Done. We have now added the accession number and more information on the *Heliconius sara* individuals used for the reference genome in Table S1. We

also provide more information on the reference genome including a link to the NCBI entry of the genome in the main text (Please see section “High-quality reference genome assembly for *Heliconius sara*”).

Response to Reviewer #2:

Rueda and colleagues have sequenced the genome of *Heliconius sara* along with 114 individuals from the sara/sapho clade. They then use this data to identify 3 sex chromosome-autosome fusions in this group. I really enjoyed reading this manuscript and believe it would be an excellent fit for PLOS Genetics. The genome assembly presented is very high quality and I found the authors' data for sex chromosome-autosome fusions to be convincing. There is much we do not understand about sex chromosome evolution and the impact of sex chromosome fusions on genome evolution, and this group of butterflies seems to be an excellent natural and diverse model clade for this area of study.

Answer: We are very happy that the reviewer enjoyed reading the manuscript and finds it to be a good fit for PLOS Genetics. We also highly appreciate the suggestions to improve our manuscript.

Despite my general enthusiasm, here are a few ways I believe the authors could improve their manuscript:

1. The increased heterozygosity shown by the authors is fairly convincing of Sex-A fusions. If the authors' are correct that this is due to lack of recombination after the Sex-A fusion event, then they should be able to support their claim by showing increased LD in females on the putative Sex-A chromosomes. Ideally this would be to phase the samples, then show that roughly half of female chromosomes display increased LD, while the other half are similar to males. If this is not possible, females should still show some increased LD, though to a lesser extent as the signal will be reduced by the unfused copy.

Answer: Thanks for your suggestion. We followed a more direct approach by applying Hi-C sequencing followed by haplotype separation (see “Evidence for three W-autosome fusions” - lines 369-403). These analyses confirmed the presence of a fused haplotype and another unfused haplotype in females (Fig. 7). The haplotypes indicated that this chromosomal rearrangement is far more complex than we had initially thought, suggesting the presence of intrachromosomal translocations and inversions.

2. Could the authors discuss their Z-A fusion model in more detail? In the case of the authors' proposed Z fusion model, I am struggling to understand how the unfused autosome would be retained in the population without acquiring W or neo-W function. For a Sex-A fusion to increase female-specific nucleotide diversity in a simple ZW sex determination system, the fused sex chromosome would need to be female-limited, so a W or a neo-W. Otherwise, the chromosome(s) would still be capable of recombination in males (due to the holocentricity of Lep chromosomes), though this could be limited to the shared PAR region/ancestral chromosome 4/9/14 if the fused sex chromosome was sufficiently diverged.

Answer: Although the new data indicates that the Sex-A fusion involves the W chromosome, we kept the figure showing the hypotheses because we believe it helps visualizing our reasoning and may be helpful for researchers that find similar patterns but only have WGS resequencing data. We have added a more detailed explanation on why a fusion with the Z chromosome can produce the patterns we observed in the population data (see lines 225-236). We now use the terms neo-Z and neo-W chromosomes for clarity purposes. If the fusions were with the Z chromosome, females would have an unfused version of 4 and 9/14 which would accumulate mutations. These could indeed be called neo-Ws and would be only found in females and thus not recombine anymore. They would remain in the population as they would align with the fused Z-4-9/14 during female meiosis and would be passed from mothers to daughters. However, now we know that fusions involve the W chromosome, and mothers pass on the unfused neo-Z chromosomes (4 and 9/14) to their sons.

3. The authors do suggest that the unfused autosome could be female specific, which would account for the missing autosomal portion of the fused chromosome in females (so, for example, a 4Z/4 genotype). This would, however, lead to the possibility of a 4Z/4 genotype without the W (if the W is not passed along), effectively making a Z0 sex determination system. Can the authors discuss this? Do they find evidence of polygenic sex determination? Are females less common than males or found in roughly equal proportion? Is a W likely present at all? Is it necessary for sex determination? To sum up, a W fusion is fairly straight forward, but a Z fusion would likely be more complicated than the authors present.

Answer: Thank you very much for these interesting questions. As mentioned earlier, we have discovered that the fusion involves the W chromosome. In this case, the Sex-A fusion is restricted to females and the fused Sex-A chromosome has accumulated mutations and/or structural variants that favoured its divergence from its unfused homolog. The unfused chromosome 4 becomes by definition a neo-Z2 chromosome in all five species of the *sapho* subclade, chromosome 9 is a neo-Z3 in *H. sapho* and *H. hewitsoni*, and chromosome 14 is a neo-Z3 in *H. congener* and *H. eleuchia* (see “Evidence for three W-autosome fusions”). Interestingly, species in the *sapho* subclade with Sex-A fusions (*H. antiochus*, *H. sapho*, *H. hewitsoni*, *H. eleuchia*, and *H. congener*) do not map to the W scaffolds of *H. sara*. This is likely due to the divergence of the W in the *sapho* subclade from that of *H. sara*, making it difficult to map reads against this reference (S2 Fig). Alternatively, the original W in the *sapho* subclade may have been lost (lines 137-148 and Fig S2). Unfortunately, there are no studies in *Heliconius* whether there is polygenic sex determination or if the W chromosome is necessary for sex determination. Although females are more difficult to capture in the wild due to their high-altitude flying behaviour, there is not significant difference in the number of females and males in the wild (Ramos & Freitas, 1999). It will be interesting to address these questions in future studies.

4. This manuscript is very descriptive without any additional analyses, which may limit its impact. Sex chromosome-autosome fusions are certainly interesting and uncommon, but not unheard of prior to this study. A very similar recent study is Flynn, Hu, and Clark (Genetics, 2023: https://doi.org/10.1093/genetics/iyad062). They do an excellent job of identifying 3 Sex-A fusions in D. virilis, validate the fusions, then show that

one of the y-autosome fusion strains demonstrates greater DNA damage response. I highlight this study because it does a nice job of investigating the consequence of sex-A fusions (to some extent), and I would encourage the authors to similarly take their study a slight step beyond identifying the three Sex-A fusions with some investigation of consequence of the fusions. This is just a suggestion (an attempt to be helpful), not a request.

Answer: We have added additional analyses that confirmed the W-autosomal fusions (see "Haplotype-based phylogenetic analysis on chromosomes 4, 9 and 14" and "Evidence for three W-autosome fusions). We agree with the reviewer that our finding calls for future analyses. To study the consequences of the fusions in greater detail, we will assemble reference genomes for all of these species in the future. However, this is a time-consuming and expensive endeavour that is outside the scope of this study. We note though, that there are not many W-autosome fusions have so far been found in Lepidoptera, whereas Z-autosome fusions seem to be quite common. It is possible that some of the W-autosome fusions were missed due to the challenge of assembling Ws.

Here are a few minor comments and suggestions:

5. I would suggest the authors use Sex-A or S-A for sex-autosome instead of SA (which often represents sexually antagonistic in the literature). Some examples of SA for sexually antagonistic: Eyer, Blumenfeld, and Vargo (2019); Ruzicka et al. (2020); Harper, Janicke, and Morrow (2020). I had to continuously remind myself that SA was sex-autosome while reading.

Answer: We have now adopted the suggested abbreviation throughout the manuscript.

6. Give a grammatical pass: E.g. 101 "One of such cases are the *Heliconius* butterflies" could have fewer words: One such case are *Heliconius* butterflies...

Answer: We have revised the entire manuscript for grammatical issues**.**

7. 135 "The ML phylogenetic tree obtained with 183,282,470 sites separated individuals into groups consistent with both PCA and ADMIXTURE analyses" should have the clause with commas: The ML phylogenetic tree, obtained with 183,282,470 sites, separated individuals into...

Answer: The sentences now state "We reconstructed a Maximum Likelihood (ML) phylogenetic tree using 183,282,470 concatenated sites. This phylogeny separated individuals into two main subclades consistent with PCA analyses" (S4 Fig) (Please see lines 167-169).

8. The authors write "83 As with other chromosomal rearrangements, SA fusions can reduce recombination and potentially strengthen reproductive isolation if they bring together barrier loci into regions with reduced recombination (12,13)". Increasing the length of the chromosomes will likely decrease recombination as well, without relying on aggregation of barrier loci. This has

been explored in Heliconiines by Martin et al. 2019 (PLOS Biology) and Cicconardi et al. 2021 (MBE) among others.

Answer: We clarified this in lines 77-78.

9. Please include the species assembled under the "Genome Assembly" subheading. I had to check the methods to identify which genome was actually assembled.

Answer: We have changed the subtitle and added more detailed information in this section for clarity purposes (see “High-quality reference genome assembly for *Heliconius sara*”).

Response to Reviewer #3:

First of all, I would like to apologize the authors for the extra time it took to send this report. Unfortunately, it was a very busy period with many duties and doing field work.

In the manuscript entitled “Three sequential sex chromosome – autosome fusions in Heliconius butterflies” the authors assembled (de novo) a high-quality reference genome for of Heliconius sara and analyzed whole genome re-sequencing data for 114 individuals of seven *Heliconius* close species (18 subspecies). The set of analysis they implemented revealed different tree topologies across some chromosomes, with different clustering patterns among individuals depending on their sex in various species. The patterns of diversity, differentiation, divergence and read depth let the claim the discovery of three sequential sex chromosome-autosome (SA) fusion events in a clade known by numerous chromosome fissions (but without any fusion described so far). While the adaptive significance of these fusions is yet to determine, understanding chromosome evolution (in particular sex chromosomes) and how that can impact in diversification is of general interest for researchers in the field. I found the paper interesting and challenging (in a positive way) but I think some clarifications and improvements are needed before publication.

1. Namely, I found the introduction about Sex-A fusions (rearrangements) and neo sex chromosomes very interesting. However, when I reached the goals at the end of page 5, they seem to be focused on the phylogenetic relationships and chromosomal rearrangements are only mention in a vague way (see details below). Thus, in my opinion goals are not enough well aligned with the rest of the introduction. If the authors want to reassess the phylogenetic relationships among species of this specific *Heliconius* species, then this problematic needs to be introduced before. However, I honestly think that a focus on the phylogeny narrows down the interest of study. Thus, my suggestion is to give more prominence on the topic of rearrangements or SA fusions in the goals.

Answer: Thank you for your suggestion. We have changed the last paragraph for clarity and revised the manuscript to have a clearer flow and stronger focus on the sex-autosome fusions.

2. I think that my main challenge is to understand whether there is enough/strong evidence for SA fusions in this system. I agree that the observation of females and males forming distinct clusters in the tree (and in the PCA/MDS) suggests the presence of regions involved in sex-determination (or sexually antagonistic selection) in those chromosomes. However, the evidence for the fusion is not direct. Despite the need for some specific clarifications (see below), I agree that the patterns are compatible with scenarios of SA fusions suggested by the authors. However, I think it would be important to do some independent validation or alternatively tone a bit done the claims (see below). I imagine the authors do not have access to long read data for the species where SA fusions could have occurred to see if they could span the chromosomes suspected to be involved in the fusion, otherwise I guess they would have done this. Alternatively, would it be possible to look at LD between SNPs across the chromosomes involved in

fusions (comparing species where fusions happened versus fusions where they did not happen)? I think this could eventually reveal physical linkage near the fusion point between one extremity of each chromosome? Perhaps phasing data to infer haplotypes would make this more feasible and informative? I must admit I do not have a better alternative. I thought about other rearrangements (like large inversions) within Chr 4, 9 and 14 but then this would not fully explain the sex-linked loci in these chromosomes, unless these inversions were involved in sex determination (neo sex chromosome evolution without fusions), but this may not be more likely. At the same time, there seem to be several taxa with chromosomes resulting from SA fusion, including H. antiochus and H. hewtsoni that maintain the ancestral number of chromosomes, which would only possible if these same species also had one and two fissions, respectively. This is possible but somehow peculiar. Again, I think the authors show an interesting scenario to explain the observed patterns. I think this is compatible with the data, despite the lack of direct evidence. Thus, if no independent validation is possible, I think I would at least tone a bit down the claims and present these as putative fusions. I think with some improvements the manuscript could eventually be published in PLoS Genetics

Answer: Thank you for your suggestions. As an independent validation, we have generated new Hi-C data for a female of *H. congener* and a male of *H. sapho* and mapped them against the reference genome of *H. sara* and *H. charithonia*. This new experimental design allowed us to conclude that the fusion is with the W chromosome. For more details, please refer to the manuscript in section "Evidence for three W-autosome fusions". We also phased the haplotypes from both the Illumina and Hi-C data (for details, refer to lines 244-264 and 610-639). Both analyses confirmed that females are heterozygous for the fusions (Fig 3 and Fig. 7). Furthermore, the haplotypes from the Hi-C data indicate that this chromosomal rearrangement is more complex than we had initially thought, suggesting the presence of possible translocations and intrachromosomal inversions. As the reviewer pointed out, *H. antiochus* and *H. hewitsoni,* which also show W-A fusions, have 21 chromosomes. These findings could be explained by two alternative scenarios: a) The chromosomal fissions in *H. eleuchia*, *H. congener* and *H. sapho* happened independently in each (sub)species after the W-A fusions and fusing to the W protected Chr4 and Chr9/14 from fissions, or b) the fissions could be ancestral, and the W-fusions could have involved the largest chromosomes that had not been broken up. In this second scenario, *H. hewitsoni* would have undergone additional autosomal fusion events restoring chromosome number to 21 after fission events. While we think the first scenario is more likely than the second one, full genome assemblies will be required to distinguish these hypotheses. We now describe this in the Discussion.

3. Detailed Comments: L58. I do not think the authors provide evidence supporting this claim.

Answer: With the new data and results we think we provide strong evidence of Sex-A fusions involving autosomes 4, 9, 14, and the W chromosome.

Introduction:

4. L106. Most readers will not be familiar with cyanogens are nor why are they important. Perhaps briefly explain.

Answer: We clarified this in lines 99-101.

5. L112. The impact of chromosomal rearrangements on what?

Answer: We clarified this in lines 104-116.

6. **Results:** L118. Please specify what species (the methods only come later). Also, in the methods it is mentioned that individual used to assemble the reference genome was a female but perhaps it could be indicated here, to help interpretation of results.

Answer: We have changed the subtitle and added additional information in this section for better clarity (see "High-quality reference genome assembly for *Heliconius sara*").

7. L123. For the sake of precision, perhaps it should also be mentioned already that BUSCO completeness was assessed using the Lepidoptera gene set.

Answer: Done.

8. L142. Remove "In this case,"

Answer: Done

9. L165-170. Not very clear. For instance, concerning Chr4, Maybe I misunderstood but I cannot see that "H. antiochus, H. sapho, H. hewitsoni, females do not group by sex among themselves" in figure 2B. Also both in H. eleuchia and H. sapho, not all females seem to group together. Please clarify. Finally, as I far as I am concerned, the authors do not present evidence showing they are "heterozygous for sex-specific haplotypes". I think the last sentence is not clear neither. A separate clade?

Answer: The reviewer is correct in assuming that the mixed signals observed in the phylogenetic trees are not very clear. This is because we conducted these analyses without separating the haplotypes, and therefore, females appear as intermediates. To separate the haplotypes, we have now phased the population data with whatsHap and shapeit4. We then used Relate to obtain SNP genealogies (see "Haplotype-based phylogenetic analysis on chromosomes 4, 9 and 14"). Additionally, we separated the haplotypes from the Hi-C data using a "Chomper" from Mackintosh et al., 2022 (see "Evidence for three W-autosome fusions"). Both analyses confirmed the presence of one fused haplotype and one unfused haplotype in females, but not in males (see figures 5 and 7). Furthermore, the haplotypes from the Hi-C data indicated that this

chromosomal rearrangement is more complex than just simple fusions, suggesting the presence of additional translocations or inversions.

10. L167. “in the former species” instead of “in these species”?

Answer: We have rewritten this paragraph.

11. L176. Is not a bit odd that the outgroup cluster with species from the H. sara clade. Actually, is it useful to include the outgroup in this PCA at all?

Answer: The reviewer is correct. We have now removed the outgroups.

12. L182-184. I think the pattern is different between H. hewitsoni and H. leucadia. Could not introgression explain the pattern for the former species?

Answer: We removed this analysis because it was not adding essential information to the manuscript.

13. L193-194. I think the MDS per se did not reveal two objective clusters (at least for Fig 3 A). If you use k-means I doubt those two clusters would be revealed. Thus, these clusters were defined I posteriori based in sex/taxonomic information. I think the procedure is fine but perhaps this needs to be reworded.

Answer: We removed this analysis because it was not adding essential information to the manuscript. Also, we were able to separate the haplotypes in both the WGS data and the Hi-C data indicating that the fusion of chromosome W occurs only with one of the haplotypes in females (see Figs 3 and 7).

14. L214-227. Would not be interesting to also calculate these parameters for each sex separately? For instance in figure 2, the branch length between each sex “haplotype” within H. hewitsoni seems lower than within H. sapho. Could this suggest a different age and thus a different fusion age and thus and independent fusion?

Answer: Thank you for your suggestion. We have calculated F_{ST} by sex and observed that it only decreases in females (see Fig. 4). We believe that results on both the heterozygosity and mean depth suggest that the fusion of the W with Chr4 is older, while the fusions with Chr9 and Chr14 are younger. (see more detailed information in the discussion).

15. L217. In Fig 4. it is H. sapho - H. hewitsoni that show the highest DXY.

Answer: We removed this information and instead added information on Fst by sex in each species.

16. L221. Lower FST than what?

Answer: We reworded this idea (see “Patterns of genetic differentiation”).

17. L221-225. These comparisons are not very clear and not very objective. I think this needs some formal testing.

Answer: We modified the F_{ST} figure (see the Fig. 4). We also calculated F_{ST} in both sexes and statistically tested that F_{ST} is lower than males and from all other chromosomes (see “Patterns of genetic differentiation”).

18. L232. Perhaps in the section starting here, it would be interesting to describe also the patterns observed for the Z chromosome. For instance, should not individual heterozygosity in females for markers in the Z chromosome be 0? It is hard to check this with standardized values. I am not sure if Figure 5 is just a hypothetical case but there is one female where the Z chromosome behave as the autosome (in terms of heterozygosity). Also, it would perhaps be interesting if the authors explain why heterozygosity is higher in males than females for autosomes. I presume is related with recombination but this is not observed in all species.

Answer: We modified this figure (see Fig. 5). Additionally, we removed the standardization and added a better explanation of this pattern in section “Sex-specific differences in heterozygosity and mean depth” and lines 329-332. Indeed, Fig. 5 (now Fig. 2) is a hypothetical case. We have improved this figure and the legend.

19. L255. Replace “H. sapho,“ by “H. sapho and”

Answer: Done.

20. L262-264. Not clear to me why depth on chromosome 4 for H. congener females is higher. I think this should be mentioned and explained because it seems to be against the hypothesis presented by the authors, right (see below)?

Answer: Unlike other species with evidence of fusions, *H. congener* showed more regions with peaks of mean depth on chromosome 4 (see Fig. 6). These peaks only are in females, suggesting the presence of repeated expansions or duplications. We clarified this in line 346-350.

21. L265-267. Actually, judging from Figure S35, not only the pattern is not the sane but it is actually the opposite, no? Any explanation for this?

Answer: Thank you for reviewing the supplementary figures. We have verified, and it was an error in generating the graph. Indeed, the mean depth pattern of chromosomes 9 and 14 is not the same as that of chromosome 4, but the reverse is not the case. This figure was modified (see Fig S37), and the discussion was changed accordingly (see lines 435-440).

22. L282. Could this suggest duplications?

Answer: Thank you for the suggestion. We fully agree that repeat expansions or duplications are expected and we do indeed see this. We have clarified this in lines 346-350.

23. L286-287. I think this also happens (but in a smaller region) for *H. sapho*. Additionally, perhaps is relevant to mention that his happens without changes in mean depth.

Answer: Thank you very much for noting it. We have added this information in lines 357-361.

24. L292. But if reads become unmappable, how can the authors access heterozygosity (or lack of it)?

Answer: In some regions the haplotype fused with the W cannot be mapped, and those regions exhibit high heterozygosity. Of course, the unfused or neo-Z haplotype continues to map, and therefore, heterozygosity or lack thereof can be calculated. This has been clarified in the text at lines 366-368.

25. L295,296. I am not sure the authors found evidence for the fusion or evidence compatible with the fusion of three autosomes to sex chromosomes.

Answer: Thank you very much for your feedback. We conducted field work and caught new butterflies and generated Hi-C data for a female of *H. congener* (suspected Sex-A fusion with 4+14) and a male of *H. sapho* (suspected Sex-A fusion with 4+9). We mapped the new Hi-C data against our *H. sara* reference genome. This revealed that the fusions are heterozygous in females, do not involve the Z chromosome, and are absent in males. All three fusions thus likely involve the W chromosome (see “Evidence for three W-autosome fusions”).

26. L316. Again, would not a more cautionary description be more appropriate?

Answer: We agree. Our new data shows the fusion involves the W chromosome.

27. L323-332. I think it would be important that in this section the authors described the series of events according to their hypothesis. This the link between fusions and patterns is explained in figure 5. But maybe in figure 6 it should be clear that two consecutive fusions may have occurred in some species. According to the scenario presented I think that H. sapho has a sex chromosome that results from two fusions, a first fusion between a Z or a W and chromosome 4 and a fusion between the result of this fusion and chromosome 9? Is this what the authors advocate. Although possible, it is striking that some species (such as H. hewitsoni and H. antiochus) and still present 21 chromosome pairs, despite the fusions? The same number of fissions should have occurred for them to maintain the same number of ancestral of chromosomes (21)?

Answer: We have modified the text and provided a better explanation of the fusion events we found (Please see Discussion). The chromosome numbers presented in Fig. 1 stem from Brown et al. 1992 (reference 36). Most of these chromosome

counts are from male gonads and thus would not show the fusions as the fusions are with the W as we know now.

Methods:

28. L381. Perhaps it would be good to provide the link for Lepbase.

Answer: We have rewritten this section (see “Genome assembly of *Heliconius sara*” in lines 514-554).

29. L385-390. Connect this section with figure 1 so that readers can know the geographic location of the samples in the map?

Answer: Done.

30. L406. Is this quality for base call? Mapping? Genotype?

Answer: It is per site. This was clarified (see lines 575-576)

31. L410-411. How was the excess of heterozygosity evaluated? Within each species?

Answer: We computed heterozygosity values for each site across all individuals in the dataset to remove regions where all individuals are heterozygous, indicating that they are reads from paralogous regions that are collapsed in the reference genome. These sites were removed from the vcf file. We have now clarified this in the manuscript.

32. L412-414. It would be good to know a little bit more about what this script does.

Answer: This script takes a VCF file as input, processes genotype data for each individual, and generates plots to visualize the allelic balance at heterozygote positions in a separate page for each individual. This file can then be used to detect contaminated individuals or barcode switching problems. Given that we did not find contamination or other issues and thus did not really use the script in a way that affected the results of our manuscript, we now removed the information in the manuscript.

33. L444. Why 6? The total number of species?

Answer: We removed this analysis because it was not adding essential information to the manuscript.

Figures:

34. Figure 2B. Should not the branch other than the blue of H. hewitsoni be red?

Answer: Yes, the reviewer is correct. It is an error in the production of the image. This was fixed.

35. Figure 3B. Not sure there is a solution for the overlap between individuals. Would smaller symbols help?

Answer: We removed this analysis because it showed a mixed signal on the clustering of individuals both by sex and species. Instead, we added the SNP-based haplotype genealogy analysis (see “Haplotype-based phylogenetic analysis on chromosomes 4, 9 and 14”).

36. Figure 4. I reported some incongruences of patterns in the main text compared with what is seen in the figure for each species pair. Please double check if all is fine with the reported values for each species comparison.

Answer: We modified this figure (see Fig. 4). The Fst was calculated by sex and we removed Dxy.

37. Figure 5. asterisks in the central plot are shifted to the left.

Answer: Thank you. We fixed this.

38. Figure 6. Are the asterisks representing significant cases of chromosomes that differ from genome average or that differ between males and females? Maybe I missed it but I could not find in the methods how the standardization of mean depth was done. Do values of -6 represent 6x times less coverage than standard? Also, would not the expectation for the Z chromosome in females be half of what is observed in the autosomes? I think it is not very clear.

Answer: We modified this figure (see Fig. 5). We also have removed the standardization and added a better explanation of this pattern in section “Sex-specific differences in heterozygosity and mean depth” and lines 329-332.

Supplementary figures:

39. Unless I misunderstood, I think it is difficult to explain mean depth differences between males and females in Figure S35 versus figure S38. Please double check.

Answer: We thank the reviewer for carefully checking the supplementary material. We have reviewed these figures and found an error in Fig S35, which has been fixed.

40. Fig S28. C) is not explained

Answer: As mentioned before, we removed this analysis because it showed a mixed signal on the clustering of individuals both by sex and species.

41. Figure S29. The distance between the two sexes within H. hewitsoni is lower than between H. sapho subspecies. Could this suggest a different age for the fusion (and thus an independent fusion)

Answer: As mentioned before, we removed this analysis because it showed a mixed signal on the clustering of individuals both by sex and species.
